# The Multifaceted Role of lncRNA GAS5: A Pan-Cancer Analysis of Its Diagnostic, Prognostic, and Therapeutic Potential

**DOI:** 10.7759/cureus.77527

**Published:** 2025-01-16

**Authors:** Asim Eisa, Safa Yousif, Mustafa A Abo-Alella, Shima Awad, Hind Tarig I Yousif, Tasneem Dafaalla, Akif Dldom, Mohamed Alfaki

**Affiliations:** 1 Genetics, University of Bahri, Khartoum, SDN; 2 Internal Medicine, University of Bahri, Khartoum, SDN; 3 Pathology and Laboratory Medicine, University of Bahri, Khartoum, SDN; 4 Epidemiology and Public Health, Tropical Medicine Research Institute, National Center for Research, Khartoum, SDN; 5 Internal Medicine, Nile Valley University, Khartoum, SDN; 6 Pediatrics, Karary University, Omdurman, SDN; 7 Laboratory Medicine, University of Port Sudan Ahlia, Port Sudan, SDN; 8 Research, Sidra Medicine, Doha, QAT

**Keywords:** bioinformatics, biomarker, cancer, expression network, gas5, lncrna, pan-cancer analysis

## Abstract

Introduction: Cancer is a significant global health problem characterized by increased incidence and large disparities in outcomes. There is a compelling need to identify novel biomarkers to enhance early detection, prognosis, and tailored therapies. Growth arrest-specific transcript 5 (GAS5) is a long noncoding RNA (lncRNA) with potential as a tumor suppressor in a subset of cancers. Its roles in the diagnosis, prognosis, and therapy across cancer types remain underexplored.

Methods: In this study, a pan-cancer comprehensive analysis of the expression status of GAS5 was conducted using various bioinformatics tools and genomic datasets, including Tumor Immune Estimation Resource (TIMER), Gene Expression Profiling Interactive Analysis (GEPIA), University of Alabama at Birmingham Cancer Data Analysis Portal (UALCAN), and Gene Expression Omnibus (GEO). The expression of GAS5 in different types of cancers was determined and then evaluated for its correlation with clinicopathological parameters, immune cell infiltration, survival outcome, and the methylation status of its promoter. Additionally, the Kaplan-Meier plotter (KMP) was used for overall survival assessment, and the cBioPortal tool was applied for genetic alteration and radiation therapy response assessment. Functional analyses were performed using data from the Long Noncoding RNA Cancer Arrays (lnCAR) database, which included coexpression networks, competing endogenous RNA (ceRNA) interactions, and pathway enrichment.

Results: Our analysis, based on three large datasets, showed that GAS5 was significantly upregulated mainly in cholangiocarcinoma (CHOL), kidney renal clear cell carcinoma (KIRC), and liver hepatocellular carcinoma (LIHC) (p < 0.05). On the contrary, it was downregulated in breast invasive carcinoma (BRCA), kidney chromophobe (KICH), and uterine corpus endometrial carcinoma (UCEC). High GAS5 expression was associated with poor overall survival in LIHC and KIRC (p < 0.05). Promoter hypomethylation was identified as a key regulatory mechanism in CHOL, KIRC, and LIHC (p < 0.05). GAS5 expression exhibited positive correlations with immune cell infiltration (e.g., CD4+ T cells, CD8+ T cells, and macrophages) in LIHC and negative correlations (e.g., B cells, dendritic cells) in KIRC. Our network analysis has established GAS5 as a ceRNA that interacts with 36 miRNAs and 95 protein-coding genes, affecting pathways like metabolism, mitogen-activated protein kinase (MAPK) signaling, and cytokine-cytokine receptor interactions in LIHC (p < 0.05). The genetic alterations may have an impact on GAS5 expression levels and how it functions during radiation treatment. Furthermore, the epigenetic control of GAS5 by DNA methylation suggests possible targets for personalized treatment methods.

Conclusion: This comprehensive analysis across various cancers offers a significant foundation for GAS5 as a potential diagnostic and prognostic biomarker, especially prominent in LIHC, with additional relevance in KIRC. Identification of tissue-specific regulatory mechanisms and correlations with immune cells opens new perspectives into the context-dependent functionality of GAS5. These findings highlight the potential of GAS5 for developing targeted therapies and advancing personalized medicine, paving the way for future research into GAS5-based treatment strategies in cancer.

## Introduction

Cancer remains a global health challenge, with rising incidence rates and significant barriers to early detection and treatment. Although advances in prevention and treatment have contributed to declining mortality rates, these gains are threatened by rising cases of cancers such as breast, pancreatic, uterine corpus, prostate, liver, kidney, and melanoma. Notably, cervical and colorectal cancers are increasing in younger populations. Colorectal cancer is now a leading cause of cancer-related deaths in individuals under the age of 50 years old [1]. While improvement in surgical techniques, chemotherapy, and radiotherapy is noted, survival disparities are observed owing to race, ethnicity, and socioeconomic status. Patients from socioeconomically disadvantaged areas face worse outcomes, with a 28% increased risk of mortality when compared to their more affluent counterparts, even when they are under high-quality care. They indicate the pressing need for innovative diagnostic and prognostic biomarkers in reducing therapeutic ineffectiveness and disparities by means of increasing the possibility of earlier detection and facilitating individually tailored therapies [2,3]. Long noncoding RNAs (lncRNAs) represent a subclass of noncoding RNAs that have increasingly emerged as important regulators of gene expression and players in carcinogenesis. A variety of lncRNAs has been identified to play an important role in the biology of tumors with recent advances in high-throughput sequencing technologies [4]. Of note, growth arrest-specific transcript 5 (GAS5) is considered a tumor-suppressor lncRNA whose expression is downregulated in various cancers, including non-small cell lung carcinoma (NSCLC), breast carcinoma, and hepatocellular carcinoma (HCC) [5,6]. GAS5 regulates genes with functions implicated in proliferation, apoptosis, and metastasis by functioning at multiple levels [7]. GAS5 functions as a competing endogenous RNA (ceRNA) or molecular sponge for oncogenic microRNAs (miRNAs), such as miR-21, implicated in several cancers. GAS5 sponges miR-21 to suppress oncogenic signaling pathways and facilitate tumor suppression [5]. In addition, GAS5 controls critical signaling pathways like PI3K/AKT/mTOR, which are fundamental to cancer progression [8]. Furthermore, GAS5 can enhance radiosensitivity by modulating DNA damage responses or influencing the cellular process in the tumor microenvironment. The overexpressed GAS5 has increased G2/M cell cycle arrest with unrepaired DNA damage and thus makes cancer cells more sensitive to ionizing radiation [9]. This makes it a promising target for radiotherapy enhancement. In addition, in the immune regulatory functions, it enhances the cytotoxic activity of natural killer (NK) cells by secreting interferon-gamma (IFN-γ) and inhibits epithelial-mesenchymal transition (EMT), which is an important event of cancer metastasis [10]. Reducing chemoresistance by targeting signaling pathways highlights its therapeutic potential [11]. Although the expression of GAS5 has been investigated in a number of human cancers, such as breast cancer and lung cancer, its pan-cancer expression pattern is not well-characterized. Based on the comprehensive literature review, only a few studies have discussed the diagnostic, prognostic, and therapeutic potentials of GAS5 in various malignancies. Thus, the present study tries to fill this knowledge gap with the aid of bioinformatic tools and genomic datasets.

## Materials and methods

This study is exploratory and descriptive, leveraging large-scale genomic datasets and advanced bioinformatics tools to analyze gene expression patterns, their correlations with clinical parameters, and potential biological functions. Our objective is to provide a comprehensive understanding of GAS5's role across various cancers while identifying its potential as a biomarker for diagnosis, prognosis, and therapeutic targeting.

Tumor Immune Estimation Resource (TIMER) database

We used TIMER (https://cistrome.shinyapps.io/timer/) to analyze the expression of GAS5 in 16 cancers and its correlation with the levels of immune cell infiltration [12]. TIMER provides an abundance of estimates for six types of immune cells: B cells, CD4+ T cells, CD8+ T cells, neutrophils, macrophages, and dendritic cells. It performs Spearman's correlation analysis to find the associations of GAS5 expression with immune cell infiltration. Statistical significance was considered at p < 0.05. Tumor purity was also included as a confounding variable in partial correlation analysis.

Gene Expression Profiling Interactive Analysis (GEPIA) database

GEPIA is a web-based tool for gene expression analysis based on data from The Cancer Genome Atlas (TCGA) and the Genotype-Tissue Expression (GTEx) projects [13]. GEPIA (http://gepia.cancerpku.cn/index.html) was used to assess the expression of GAS5 in tumors and normal tissues of various types of cancers. Through the "Expression Analysis BoxPlot" module, differential expression profiles were generated using thresholds of p < 0.05 to define statistical significance and log2|fold change| ≥ 1 to define the differential expression.

University of Alabama at Birmingham Cancer Data Analysis Portal (UALCAN) database

We used the UALCAN (http://ualcan.path.uab.edu) to validate GAS5 expression levels in various cancers and assess their relationship with clinical parameters such as age, gender, race, and cancer stage [14]. We also assessed the level of methylation in the promoter region of GAS5 using β values ranging from 0 (unmethylated) to 1 (fully methylated). High methylation was defined as β values in the range of 0.5-0.7, while the hypomethylation state was defined as β values < 0.25. The survival analysis was performed with the log-rank test, where p < 0.05 was considered significant.

Kaplan-Meier plotter (KMP) database

The KMP (https://kmplot.com/analysis/) was used to analyze the prognostic value of GAS5 expression in several cancers by comparing overall survival (OS) between the high- and low-expression groups [15]. This web tool merged data from more than 35,000 samples and 21 tumor types. Kaplan-Meier survival curves were generated in the present study for those types of cancer for which GAS5 showed significant differential expression in other databases. Statistical significance was assessed using the log-rank test (p < 0.05).

cBioPortal database

We used cBioPortal for Cancer Genomics (https://www.cbioportal.org/) to explore genetic alterations in GAS5 across 32 TCGA studies comprising 10,967 tumor samples [16]. Types of mutations and their frequencies, possible effects on overall survival and GAS5 expression, and available radiation therapy status of the cancers were analyzed.

Gene Expression Omnibus (GEO) database

The GEO was used to validate the expression levels of GAS5 with publicly available microarray datasets. We performed differential expression analysis using the GEO2R tool (https://www.ncbi.nlm.nih.gov/geo/geo2r) [17], under the criteria |log2FC| > 1 and adjusted p < 0.05. Differentially expressed genes (DEGs) were visualized by volcano plots generated via the SRplot platform (https://www.bioinformatics.com.cn/srplot), an analytical and visualization tool for DEGs [18].

Long Noncoding RNA Cancer Arrays (lnCAR) database 

The lnCAR (https://lncar.renlab.org/) is a comprehensive genomic repository of lncRNAs associated with various cancer arrays [19]. In the present study, lnCAR was used for the enrichment analysis of Kyoto Encyclopedia of Genes and Genomes (KEGG) pathways, coexpression networks, and the interaction between microRNAs and protein-coding genes in a ceRNA network, with a focus on LIHC in particular. We prioritized liver hepatocellular carcinoma (LIHC) due to the availability of more data compared to other cancers. One dataset, LI_S532, from the GEO database (GSE6764), was selected for further analysis due to the reason of comprehensive molecular profiling and highly differential expression of GAS5 in LIHC with logFC 1.5670. This dataset examined 75 tissue samples of different stages of hepatitis C-induced liver cancer using the Affymetrix U133 microarray platform [20]. GAS5 expression in this dataset was validated by using quantitative real-time polymerase chain reaction (PCR) on the Plus 2.0 platform.

Statistical analysis

A variety of statistical methods was carried out to evaluate the expression of GAS5 and its correlations in diverse cancers. The Wilcoxon test, as implemented in the TIMER platform, was performed to compare the expression levels of GAS5 between tumor and normal tissues. The one-way analysis of variance (ANOVA) in GEPIA was used to compare the expression of GAS5 between pathological stages. Welch's t-test in UALCAN was utilized to compare the expression between subgroups of patients according to clinical parameters such as age, gender, and race. The log-rank test was performed for survival analysis to demonstrate the difference in overall survival between the high- and low-GAS5 expression groups, with a threshold of p < 0.05. Partial Spearman's rank correlation analysis was performed in order to determine associations between GAS5 expression and the levels of immune cell infiltration, taking into account tumor purity as a confounder. The Mann-Whitney U test was performed to compare GAS5 mRNA expression z-scores between patients on radiation therapy and those without radiation therapy.

## Results

Analysis of GAS5 expression across multiple cancer types

**Figure 1:**
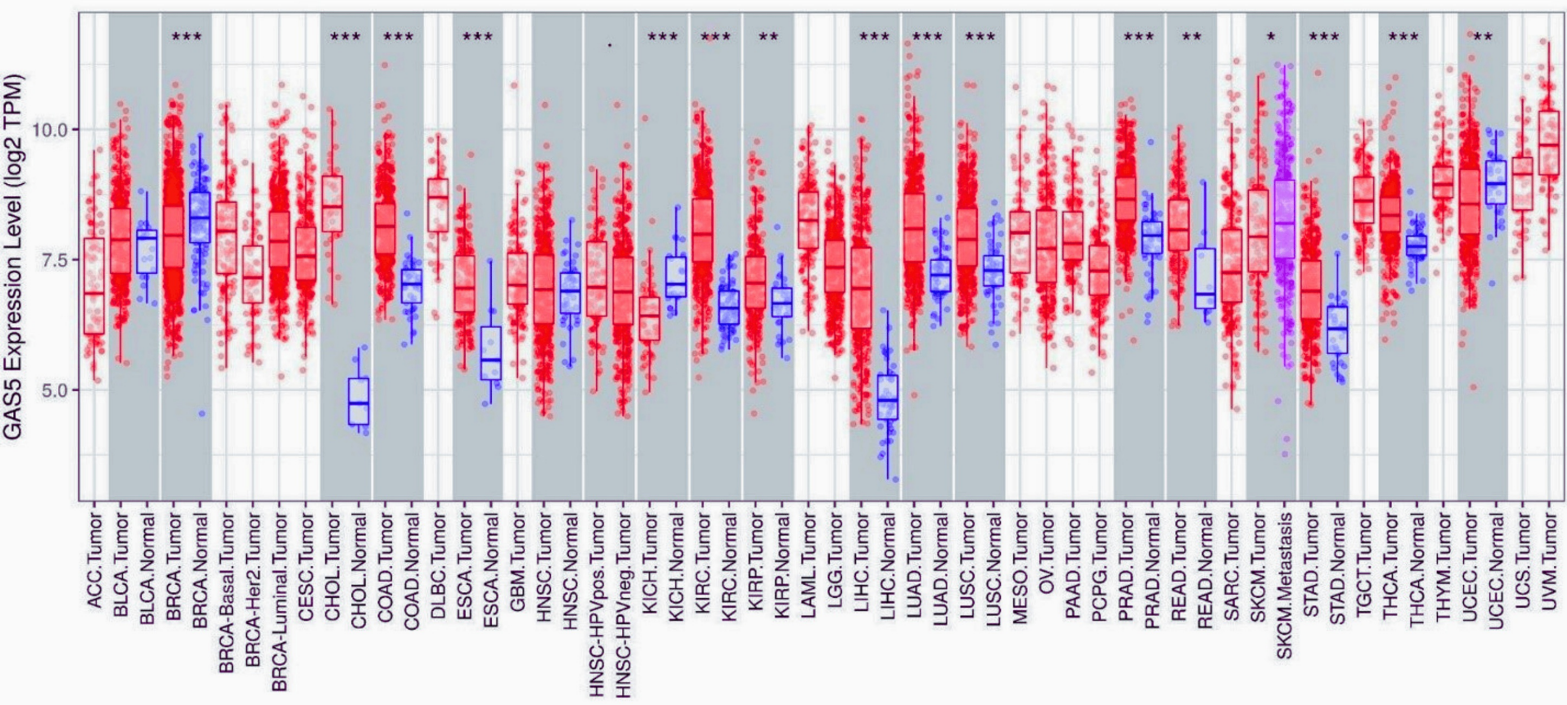
GAS5 expression levels across multiple cancer types and normal tissues using TIMER database GAS5: growth arrest-specific transcript 5; TPM: transcripts per million; TCGA: The Cancer Genome Atlas; TIMER: Tumor Immune Estimation Resource; ACC: adrenocortical carcinoma; BLCA: bladder urothelial carcinoma; BRCA: breast invasive carcinoma; CHOL: cholangiocarcinoma; COAD: colon adenocarcinoma; DLBC: diffuse large B cell lymphoma; ESCA: esophageal carcinoma; HNSC: head and neck squamous cell carcinoma; KICH: kidney chromophobe; KIRC: kidney renal clear cell carcinoma; KIRP: kidney renal papillary cell carcinoma; LIHC: liver hepatocellular carcinoma; LUAD: lung adenocarcinoma; LUSC: lung squamous cell carcinoma; OV: ovarian serous cystadenocarcinoma; PAAD: pancreatic adenocarcinoma; PRAD: prostate adenocarcinoma; READ: rectum adenocarcinoma; SARC: sarcoma; SKCM: skin cutaneous melanoma; STAD: stomach adenocarcinoma; TGCT: testicular germ cell tumors; THCA: thyroid carcinoma; UCS: uterine carcinosarcoma; UCEC: uterine corpus endometrial carcinoma; UVM: uveal melanoma The box plot shows the differential expression of GAS5  across multiple cancer types (red) and corresponding normal tissues (blue). The y-axis represents log2 TPM  expression values. Cancer types are indicated on the x-axis using TCGA nomenclature. Statistical significance is denoted by asterisks (***p < 0.001, **p < 0.01, *p < 0.05)

In order to obtain a general understanding of the differential expression of GAS5 between tumor and normal tissues, we employed the TIMER database to examine the expression level of GAS5 in 16 distinct cancer types (Figure 1). GAS5 was substantially upregulated in 13 cancer types, including cholangiocarcinoma (CHOL), colon adenocarcinoma (COAD), esophageal carcinoma (ESCA), kidney renal clear cell carcinoma (KIRC), kidney renal papillary cell carcinoma (KIRP), LIHC, lung adenocarcinoma (LUAD), lung squamous cell carcinoma (LUSC), prostate adenocarcinoma (PRAD), rectum adenocarcinoma (READ), metastatic skin cutaneous melanoma (SKCM metastasis), stomach adenocarcinoma (STAD), and thyroid carcinoma (THCA). Interestingly, compared to nonmetastatic tumors, GAS5 expression was significantly higher in metastatic SKCM. On the other hand, GAS5 was significantly downregulated in three types of cancer, which include breast invasive carcinoma (BRCA), kidney chromophobe (KICH), and uterine corpus endometrial carcinoma (UCEC). These findings shed light on the potential role of GAS5 as a biomarker in distinguishing malignant tissues from normal ones in different malignancies.

**Figure 2:**
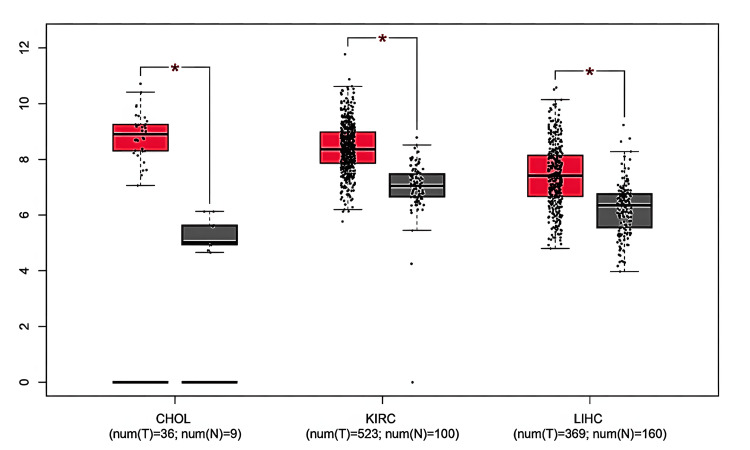
GAS5 expression levels across specific cancer types using GEPIA database GAS5: growth arrest-specific transcript 5; GEPIA: Gene Expression Profiling Interactive Analysis The box plots demonstrate differential expression patterns between tumors (red boxes) and normal tissue samples (gray boxes) across three cancer types: cholangiocarcinoma (CHOL), kidney renal clear cell carcinoma (KIRC), and liver hepatocellular carcinoma (LIHC). The y-axis represents expression levels (log2 scale), while individual data points show sample-specific values. Sample sizes are indicated for each group (n). Statistical significance is denoted by asterisks (*p < 0.05), and differential expression was determined using |Log2FC| Cutoff (FC) ≥ 1

Using the GEPIA database, we further analyzed GAS5 expression in the 16 malignancies identified by TIMER. We found that GAS5 was significantly upregulated in CHOL, KIRC, and LIHC compared to normal tissues (Figure 2). These results confirm the analysis from TIMER and again highlight the possible diagnostic relevance of GAS5 in these particular cancer types.

**Figure 3:**
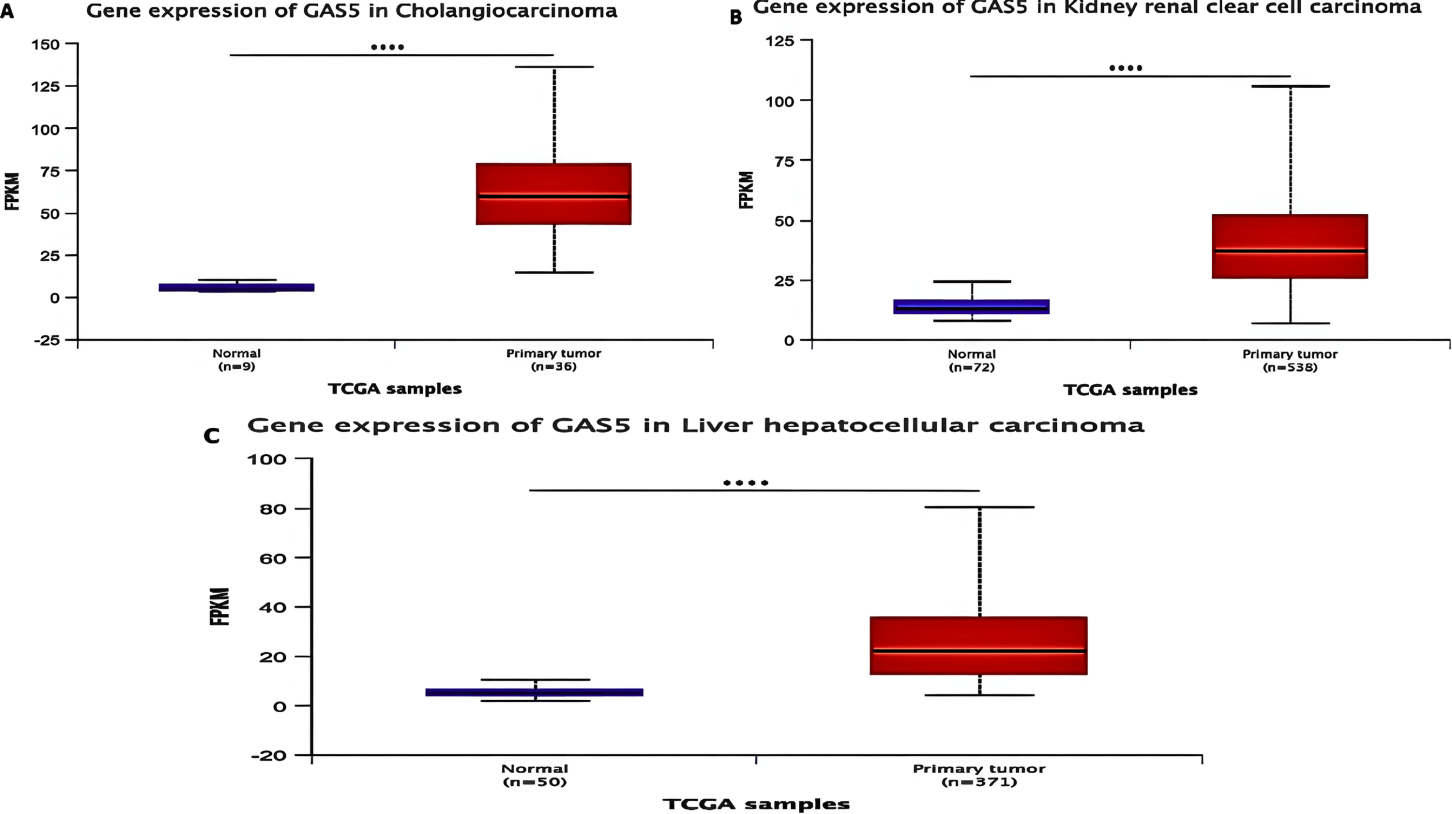
GAS5 expression levels across specific cancer types using UALCAN database GAS5: growth arrest-specific transcript 5; UALCAN: University of Alabama at Birmingham Cancer Data Analysis Portal; TCGA: The Cancer Genome Atlas The box plots compare GAS5 gene expression between normal and tumor tissues across three cancer types: (A) cholangiocarcinoma (CHOL), (B) kidney renal clear cell carcinoma (KIRC), and (C) liver hepatocellular carcinoma (LIHC). The y-axis represents gene expression levels in fragments per kilobase million (FPKM), while the x-axis shows TCGA sample categories. Blue boxes represent normal tissue samples, and red boxes represent tumor samples. Sample sizes are indicated for each group (n). Statistical significance is denoted by asterisks (**** indicates p < 0.0001)

We validated the expression of GAS5 in CHOL, KIRC, and LIHC using the UALCAN database, which supported the results from TIMER and GEPIA. According to our analysis, GAS5 was significantly upregulated in these cancers compared to normal tissues (Figure 3). Moreover, GAS5 showed downregulated trends in BRCA, KICH, and UCEC in the GEPIA and UALCAN databases, even though this finding did not show statistical significance (p > 0.05). The consistent upregulation across all three datasets (p < 0.05) highlights the potential of GAS5 as a diagnostic biomarker for CHOL (Figure 3A), KIRC (Figure 3B), and LIHC (Figure 3C). From these results, we have prioritized these three cancer types for further investigation.

GAS5 expression analysis based on clinicopathological parameters

Age-Based Analysis

**Figure 4:**
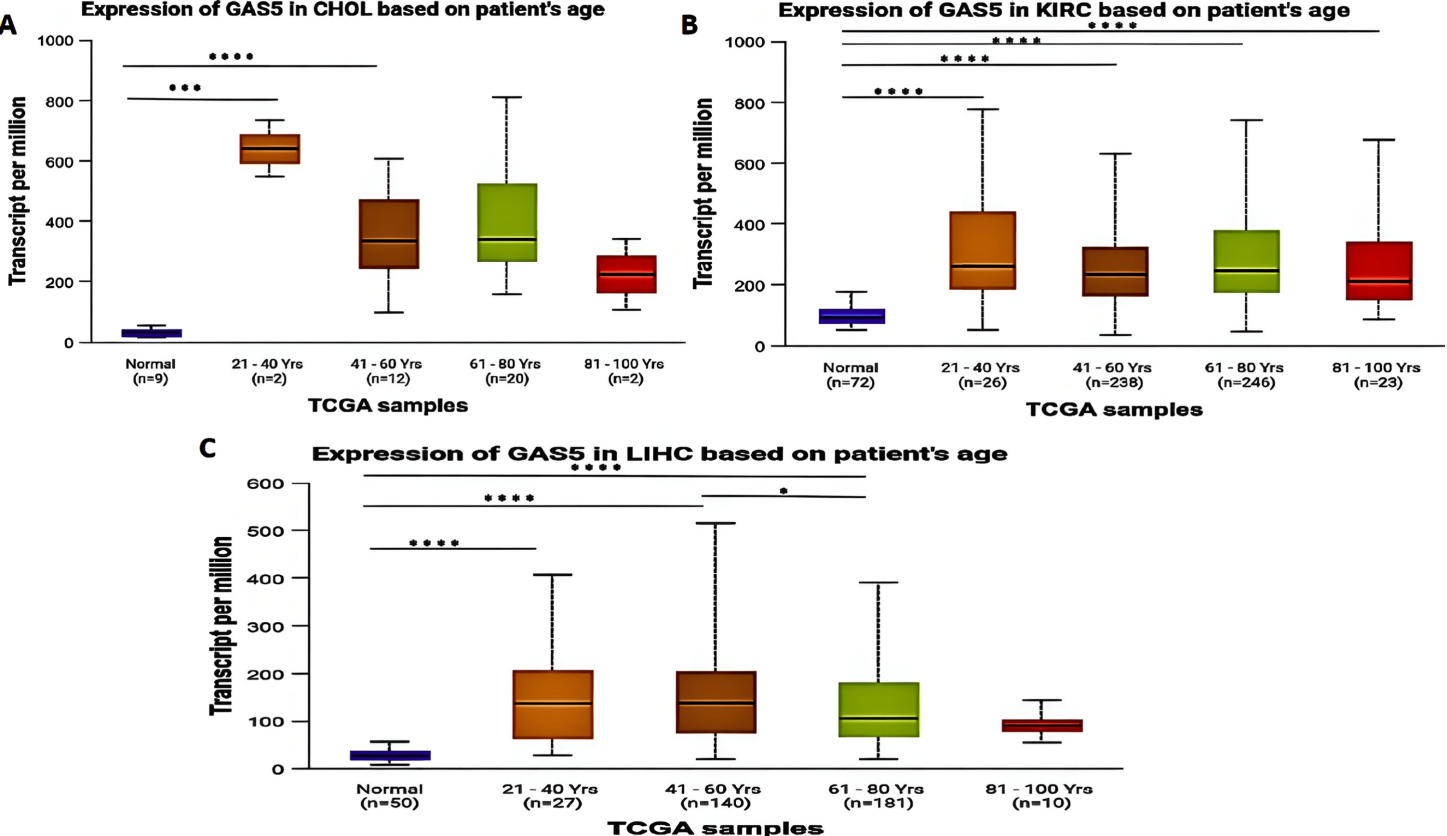
GAS5 expression in specific cancer types based on patient age using UALCAN database GAS5: growth arrest-specific transcript 5; UALCAN: University of Alabama at Birmingham Cancer Data Analysis Portal The box plots show GAS5 expression levels in (A) cholangiocarcinoma (CHOL), (B) kidney renal clear cell carcinoma (KIRC), and (C) liver hepatocellular carcinoma (LIHC) stratified by patient age groups. Expression values are presented as transcripts per million (TPM). The groups are categorized as follows: normal tissue, 21-40 years, 41-60 years, 61-80 years, and 81-100 years. Sample sizes are indicated for each group (n). Statistical significance is indicated by asterisks (****p < 0.0001, ***p < 0.001, **p < 0.01, *p < 0.05)

We categorized patients by age group (young adults: 21-40 years; middle-aged adults: 41-60 years; older adults: 61-80 years; and elderly: 81-100 years). Our analysis revealed that GAS5 was significantly upregulated in CHOL patients (Figure 4A) for middle-aged adults (p < 0.001) and older adults (p < 0.0001), but not in young adults or elderly individuals when compared with normal tissues. For KIRC patients, the expression level of GAS5 was significantly upregulated for all age groups (p < 0.0001; Figure 4B). For LIHC patients (Figure 4C), GAS5 expression was significantly upregulated in young adults (p < 0.001), middle-aged adults, and older adults (p < 0.0001). Moreover, a significant difference was found between middle-aged and older adults with LIHC (p < 0.05). On the other hand, no significant differences were observed between elderly individuals with LIHC and normal tissues. 

*Gender-Based Analysis* 

**Figure 5:**
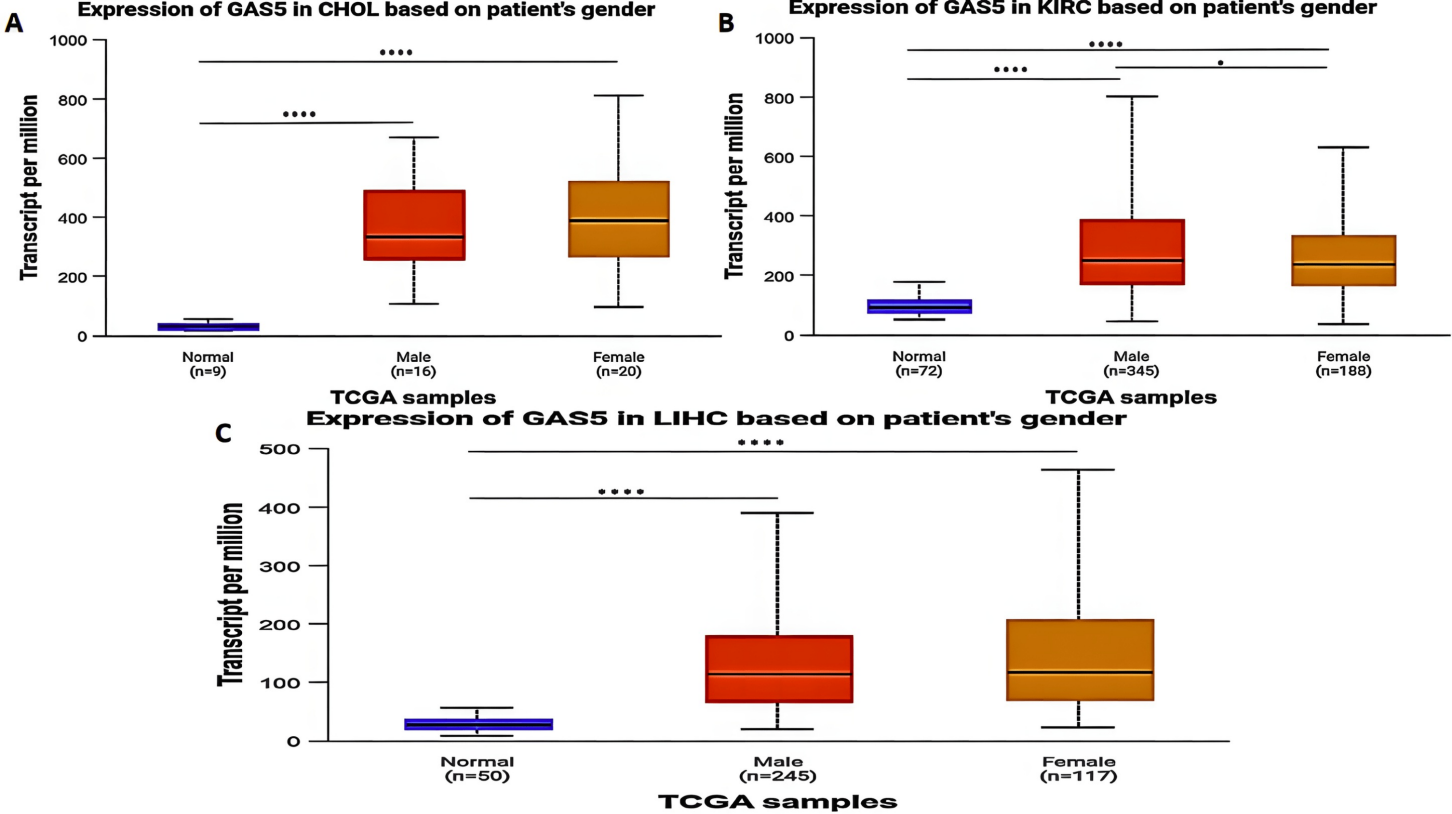
GAS5 expression in specific cancer types based on patient gender using UALCAN database GAS5: growth arrest-specific transcript 5; UALCAN: University of Alabama at Birmingham Cancer Data Analysis Portal The box plots showing GAS5 expression levels (in transcripts per million, TPM) in tumor and normal tissues across three cancer types: (A) cholangiocarcinoma (CHOL), (B) kidney renal clear cell carcinoma (KIRC), and (C) liver hepatocellular carcinoma (LIHC). The x-axis represents sample groups categorized by gender (male and female) and normal tissues, while the y-axis represents GAS5 expression levels. Blue boxes represent normal tissues, red boxes represent male tumor samples, and orange boxes represent female tumor samples. Sample sizes are indicated for each group (n). Statistical significance is denoted by asterisks (****p < 0.0001, ***p < 0.001, **p < 0.01, *p < 0.05)

The expression level of GAS5 in CHOL, KIRC, and LIHC patients was significantly upregulated when compared with normal tissues in both males and females (P < 0.0001) (Figure 5). In KIRC patients (Figure 5B), GAS5 expression was notably higher in males than in females (P < 0.05). However, no significant differences were observed between males and females in CHOL (Figure 5A) or LIHC (Figure 5C) when compared to normal tissues.

Racial-Based Analysis

**Figure 6:**
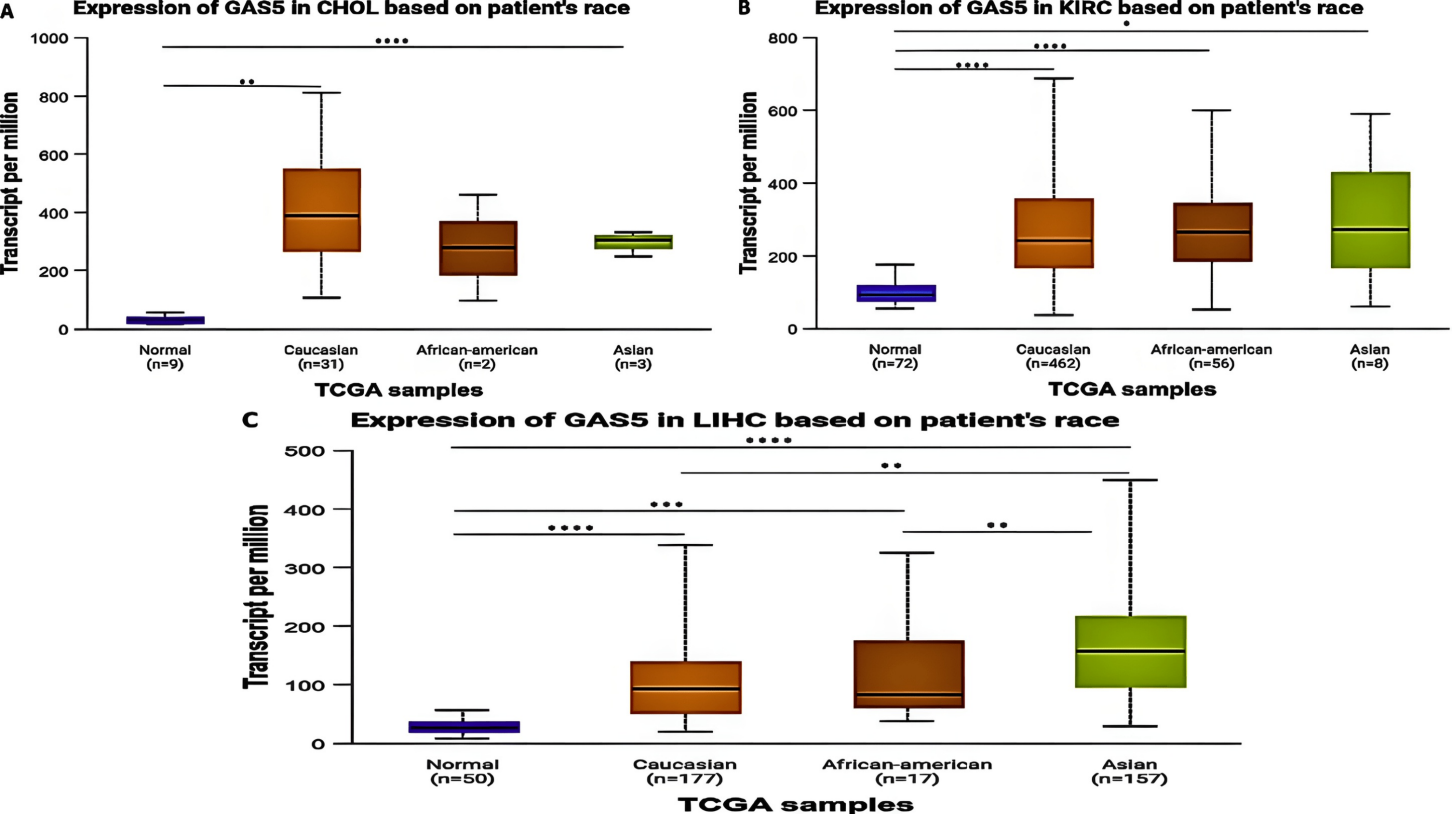
GAS5 expression in specific cancer types based on patient race using UALCAN database GAS5: growth arrest-specific transcript 5; UALCAN: University of Alabama at Birmingham Cancer Data Analysis Portal The box plots show GAS5 expression levels across different racial groups in (A) cholangiocarcinoma (CHOL), (B) kidney renal clear cell carcinoma (KIRC), and (C) liver hepatocellular carcinoma (LIHC). The y-axis represents transcript expression per million, and the x-axis shows normal tissues and different racial categories (Caucasian, African-American, and Asian). Sample sizes are indicated for each group (n). Statistical significance is denoted by asterisks (****p < 0.0001, ***p < 0.001, **p < 0.01, *p < 0.05)

The expression of GAS5 across racial groups revealed significant variability in CHOL, KIRC, and LIHC patients (Figure 6). For CHOL patients, there was a significant upregulation of GAS5 in Caucasian (p < 0.0001) and Asian (p < 0.01) patients with respect to normal controls; no significant differences were noted in African American patients (Figure 6A). No significant differences in GAS5 expression were found among the racial groups themselves. In KIRC patients, GAS5 expression exhibited significant upregulation across all racial categories. Caucasian (p < 0.0001) and African American (p < 0.0001) patients showed extremely significant upregulation compared to normal controls, while Asian patients displayed lower levels of upregulation (p < 0.05) (Figure 6B). Similar to CHOL, no significant differences were observed among the racial groups themselves. For LIHC patients, GAS5 expression was significantly upregulated in Caucasian (p < 0.0001), African American (p < 0.001), and Asian (p < 0.0001) patients compared to normal tissues (Figure 6C). Additionally, significant differences were observed between Caucasian and Asian patients (p < 0.01) as well as between African American and Asian patients (p < 0.01). 

Cancer Stages Analysis

**Figure 7:**
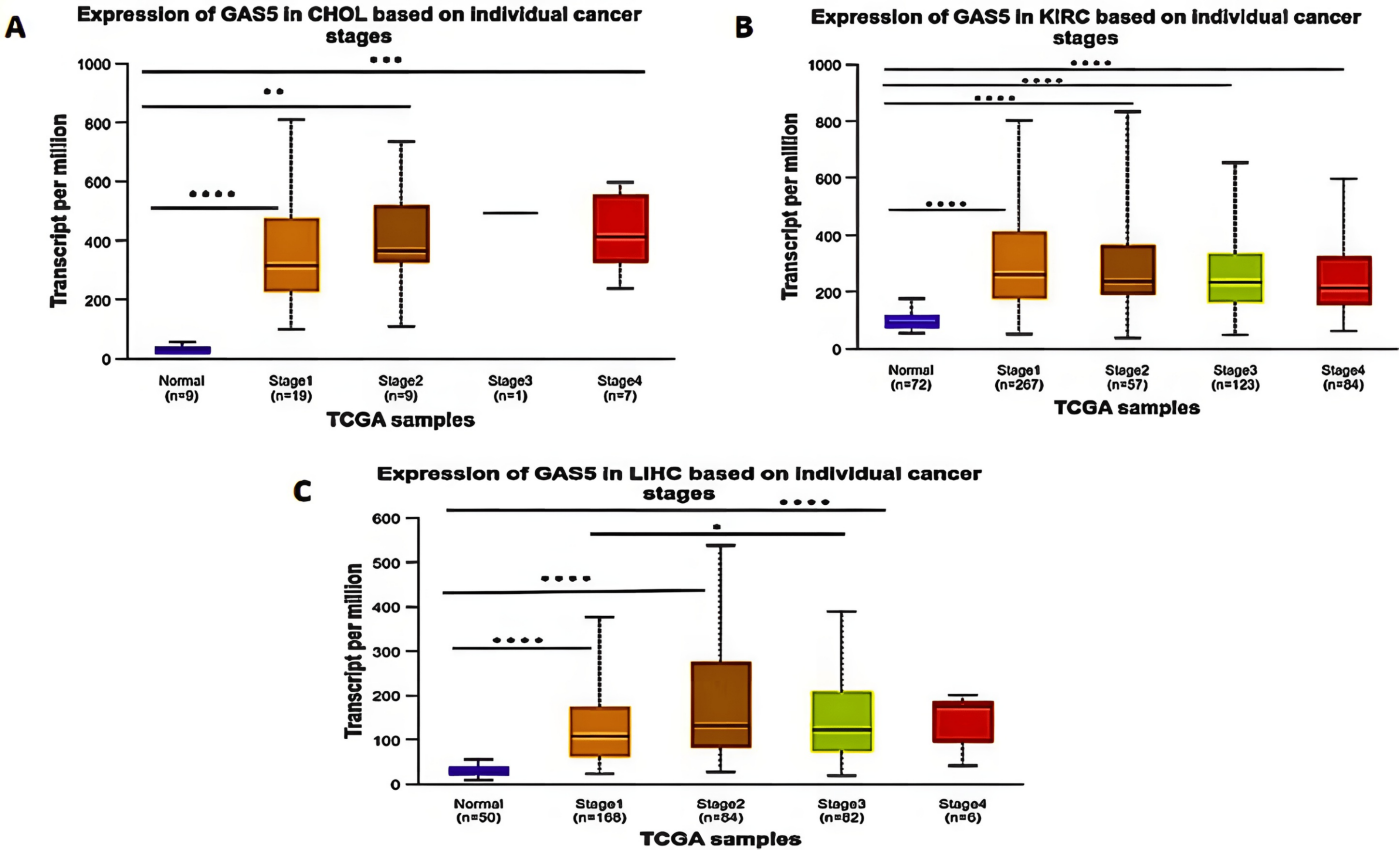
GAS5 expression across cancer stages in specific cancer types using UALCAN database GAS5: growth arrest-specific transcript 5; UALCAN: University of Alabama at Birmingham Cancer Data Analysis Portal; TCGA: The Cancer Genome Atlas The box plots compare GAS5 expression (TPM) between normal tissues and tumor tissues at individual cancer stages (Stages I-IV) in (A) cholangiocarcinoma (CHOL), (B) kidney renal clear cell carcinoma (KIRC), and (C) liver hepatocellular carcinoma (LIHC). These plots were generated using UALCAN. The y-axis represents GAS5 expression levels, and the x-axis represents TCGA sample groups, including normal tissues and tumor stages. Sample sizes are indicated for each group (n). Statistical significance is denoted by asterisks (****p < 0.0001, ***p < 0.001, **p < 0.01, *p < 0.05)

The expression of GAS5 in different stages of cancer provided us with important insights. The cancer stages were grouped by using the American Joint Committee on Cancer (AJCC) staging system, and UALCAN analysis was performed using independent t-tests comparing GAS5 expression in each cancer stage to normal tissues and between stages (Figure 7). This analysis indicated that in CHOL patients (Figure 7A), GAS5 expression was significantly higher in Stage I (p < 0.0001) compared to Stage II (p < 0.01) and Stage IV (p < 0.001) versus normal samples; however, Stage III showed no significant difference due to the limited number of samples. In KIRC patients (Figure 7B), GAS5 expression was significantly upregulated in all cancer stages versus normal samples (p < 0.0001). In LIHC patients, GAS5 expression showed significant differences between Stages I, II, and III compared with normal samples (p < 0.0001) (Figure 7C). The upregulation of GAS5 was highest in Stage IV, although this increase was not statistically significant. Furthermore, a striking difference in GAS5 expression was observed between Stage I and Stage III in LIHC (p < 0.05), which was not found in CHOL and KIRC.

Correlation analysis of GAS5 expression and infiltrating immune cells

**Figure 8:**
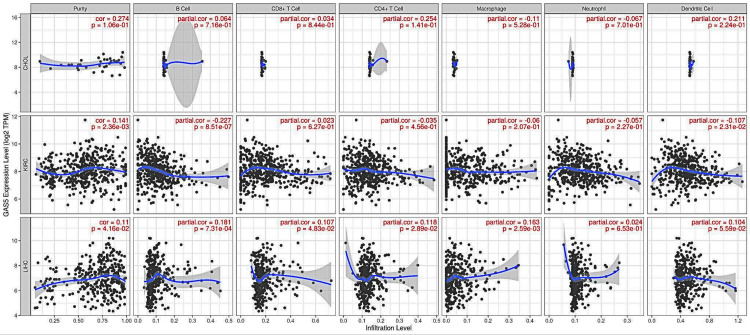
Correlation analysis between GAS5 expression and immune cell infiltration levels using TIMER database GAS5: growth arrest-specific transcript 5; TIMER: Tumor Immune Estimation Resource The scatter plots show the correlation between GAS5 expression levels (log2 TPM) and immune cell infiltration levels across three cancer types: cholangiocarcinoma (CHOL), kidney renal clear cell carcinoma (KIRC), and liver hepatocellular carcinoma (LIHC). The immune cell types analyzed include (A) purity, (B) B cells, (C) CD8+ T cells, (D) CD4+ T cells, (E) macrophages, (F) neutrophils, and (G) dendritic cells. Each dot represents an individual sample, with the blue line indicating the regression line and the grey shading showing the 95% confidence interval. The x-axis represents the infiltration level, and the y-axis shows the GAS5 expression level. Partial correlation coefficients (r) and p-values are displayed for each plot, calculated using the TIMER algorithm

The correlations between GAS5 expression and immune cell infiltration across selected cancer types were analyzed to understand the tumor immune microenvironment (Figure 8). In CHOL, the correlations between GAS5 expression and immune cell infiltration levels were generally weak and not statistically significant. In KIRC, GAS5 expression showed a statistically significant positive correlation with tumor purity (r = 0.141, p = 0.00236), indicating a weak positive association. Conversely, GAS5 expression exhibited statistically significant weak negative correlations with immune cell infiltration, particularly with B cells (r = −0.227, p = 0.000000851) and dendritic cells (r = −0.107, p = 0.0231. In LIHC, GAS5 expression showed several statistically significant positive correlations with immune cell infiltration. Notably, it correlated weakly but significantly with tumor purity (r = 0.11, p = 0.0416) and B cell infiltration (r = 0.181, p = 0.000731). Weak yet significant correlations were also found between CD8+ T cells (r = 0.107 and p = 0.0483), CD4+ T cells (r = 0.118 and p = 0.0289), and macrophage infiltration (r = 0.163 and p = 0.00259. These results suggest that the correlations present between GAS5 expression and immune cell infiltrations in LIHC and KIRC are largely not strong. As such, GAS5 is most likely not strongly related to immune cell infiltrations in CHOL.

Evaluating GAS5 as a prognostic biomarker

**Figure 9:**
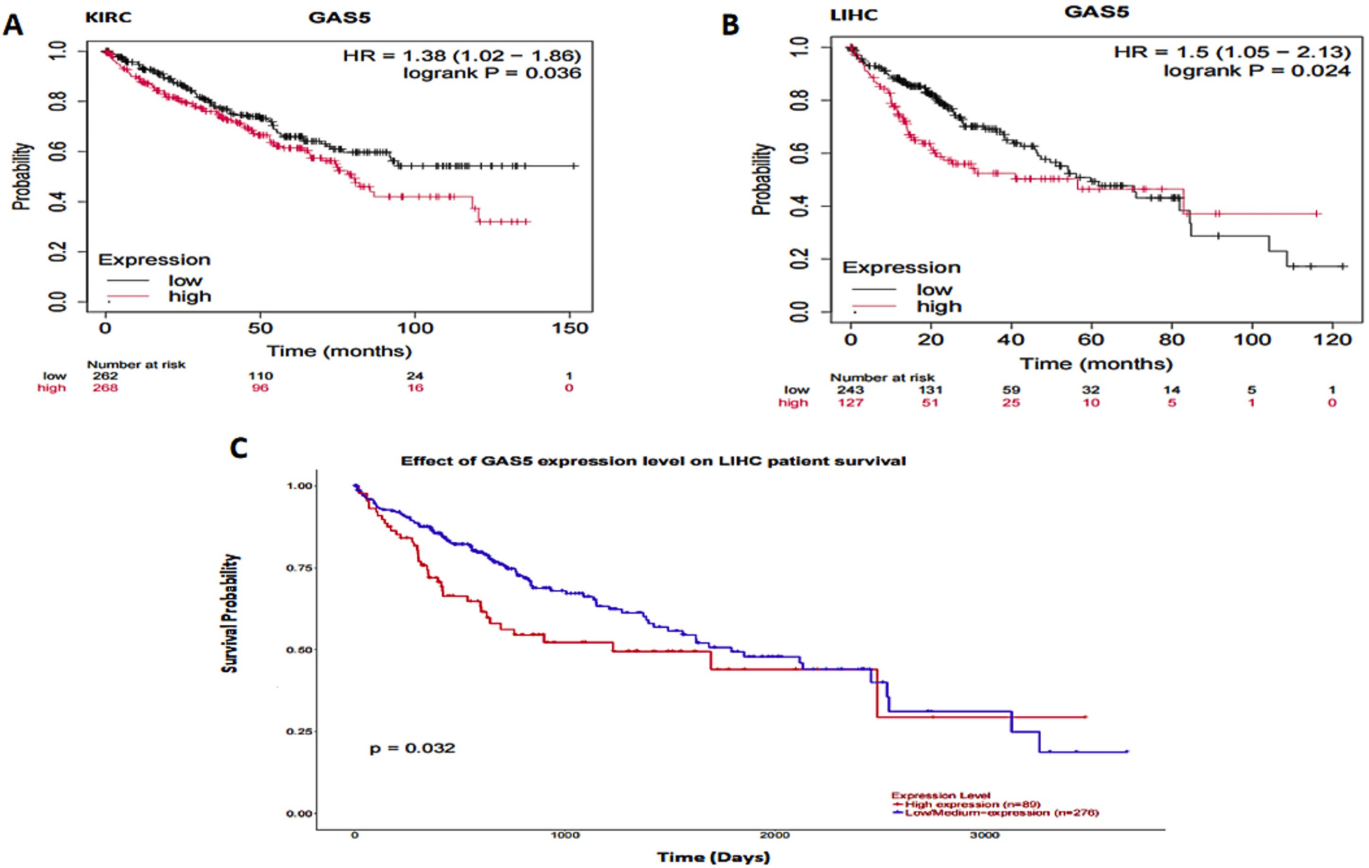
Kaplan-Meier survival analysis of GAS5 expression in specific cancer patients GAS5: growth arrest-specific transcript 5; UALCAN: University of Alabama at Birmingham Cancer Data Analysis Portal (A) Kaplan-Meier survival curve for kidney renal clear cell carcinoma (KIRC) patients, generated using the Kaplan-Meier plotter database. (B) Kaplan-Meier survival curve for liver hepatocellular carcinoma (LIHC) patients, generated using the Kaplan-Meier plotter database. (C) Survival probability curve for LIHC patients, generated using the UALCAN database. Each panel illustrates the relationship between GAS5 expression levels (low vs. high) and overall survival probability over time. Statistical significance is indicated by p-values (< 0.05), and the number of patients at risk is displayed below each curve

The relationship between GAS5 expression and overall survival was analyzed using three large databases: GEPIA, UALCAN, and KMP (Figure 9). In CHOL, neither the GEPIA nor the UALCAN datasets provided any differences in overall survival between high and low GAS5 expression groups. Additionally, the KMP presented no survival data on CHOL. In KIRC, GEPIA and UALCAN did not show significant differences in survival outcomes. The KMP (Figure 9A) showed that high GAS5 expression was associated with worse survival outcomes (HR = 1.38; p = 0.036). In LIHC, the GEPIA dataset did not show any significant differences in overall survival between the high and low-expression groups of GAS5. However, both the KMP (Figure 9B, p = 0.024, HR = 1.5) and UALCAN (Figure 9C, p = 0.032) revealed a significant association between high GAS5 expression and poorer survival outcomes. Altogether, these results showed that overexpression of GAS5 was an unfavorable prognostic biomarker for LIHC and might have predictive value in KIRC, based on the KMP data, no significant associations in CHOL were found. The mixed results across datasets call for more investigations to be carried out in order to confirm these observations.

GAS5 methylation analysis

**Figure 10:**
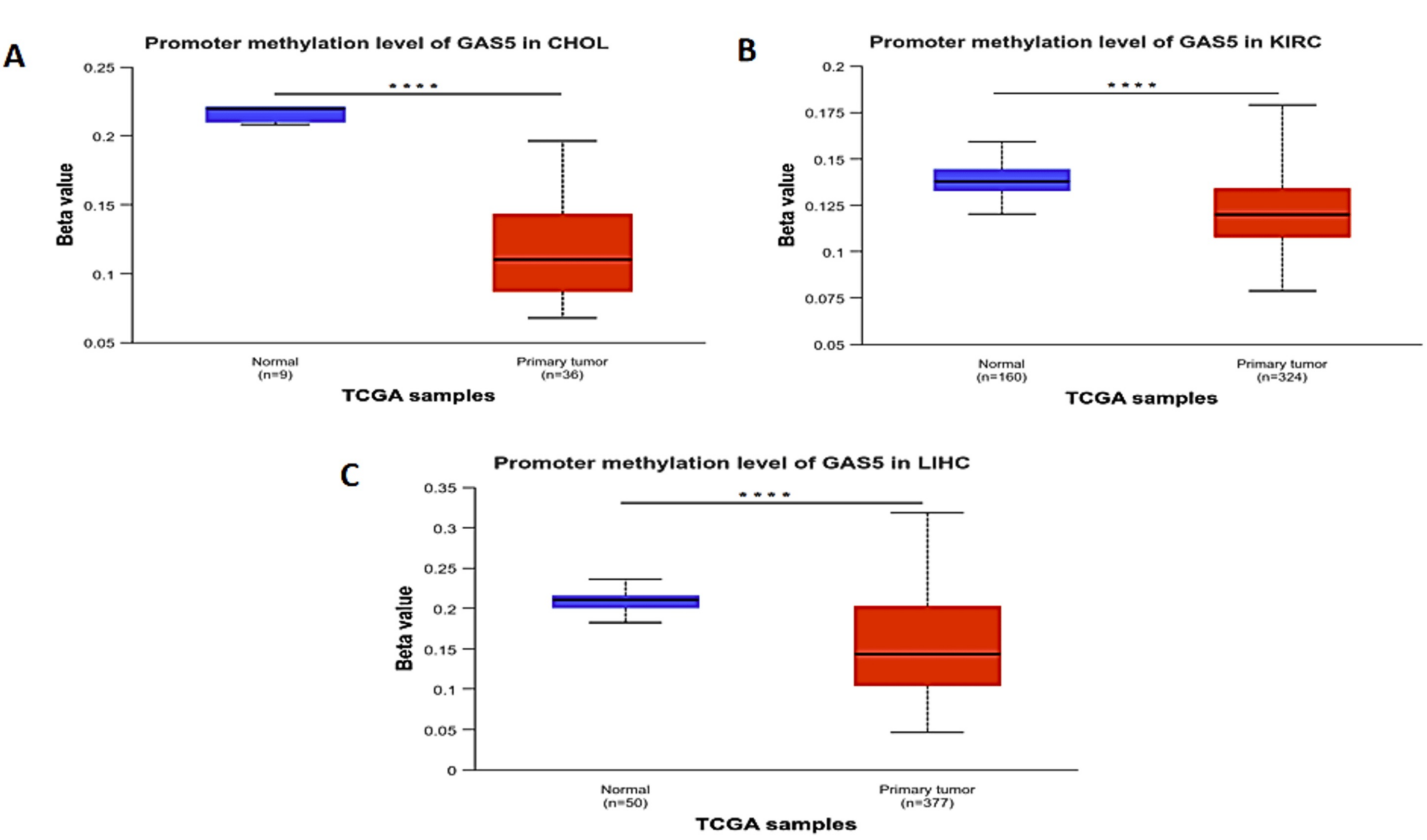
Analysis of GAS5 promoter methylation levels in specific cancer types using the UALCAN database GAS5: growth arrest-specific transcript 5; UALCAN: University of Alabama at Birmingham Cancer Data Analysis Portal (A) Cholangiocarcinoma (CHOL), (B) kidney renal clear cell carcinoma (KIRC), and (C) liver hepatocellular carcinoma (LIHC). The box plots display the beta values representing methylation levels, where higher values indicate increased methylation. Blue boxes represent normal tissue samples, and red boxes represent primary tumor samples. Sample sizes are indicated for each group (n). Statistical significance is denoted by asterisks (****p < 0.0001)

DNA methylation is a heritable epigenetic modification involving the addition of a methyl group to DNA by the action of the enzyme DNA methyltransferases. It plays a critical role in cancer and other diseases. Analysis of the methylation level of the GAS5 promoter using TCGA data in the UALCAN database showed statistically significant differences (p < 0.0001) between normal and primary tumor samples in CHOL (Figure 10A), KIRC (Figure 10B), and LIHC (Figure 10C). The methylation levels of the GAS5 promoter in the primary tumor samples in all three types of cancer maintained a steady beta value within the hypomethylation range (<0.25), indicating significantly reduced methylation levels compared with normal tissues. This trend of GAS5 promoter hypomethylation suggests a mechanism by which it may be dysregulated in these cancers.

Analysis of GAS5 genetic alterations

**Figure 11:**
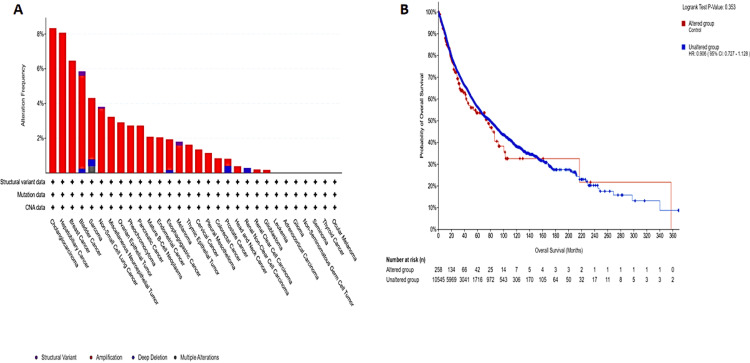
Analysis of GAS5 genetic alterations and overall survival outcomes using cBioPortal database GAS5: growth arrest-specific transcript 5 (A) Bar chart depicting the frequency and types of GAS5 genetic alterations across multiple cancer types. (B) Kaplan-Meier survival curve comparing overall survival probabilities between patients with altered GAS5 expression (red curve) and those with unaltered expression (blue curve). The x-axis represents overall survival in months, and the y-axis shows survival probability. The log-rank test p-value (p = 0.353) indicates no statistically significant difference in survival outcomes between the two groups

Genetic variation analysis of GAS5 in various tumor types using data from TCGA and cBioPortal revealed alterations in 2% of cases (259 out of 10,967 samples from 32 studies) (Figure 11A), the most frequent type of alteration being amplifications. CHOL and hepatobiliary cancers had the highest frequencies of alteration, over 7%, followed by KIRC with about 1%, and ocular melanoma and thyroid cancer with less than 1%. Bladder cancer (6%) and sarcoma (4%) showed more complex profiles with both structural variants and deep deletions. Overall survival analysis did not statistically distinguish patients with altered versus unaltered GAS5 expression (Figure 11B, p = 0.35).

Validation of GAS5 expression 

**Figure 12:**
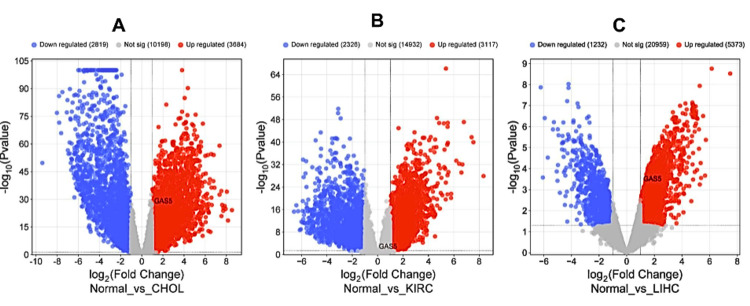
Volcano plots displaying the upregulation of GAS5 among differentially expressed genes in specific cancer types from GEO database GAS5: growth arrest-specific transcript 5; GEO: Gene Expression Omnibus (A) Volcano plot of differential gene expression in cholangiocarcinoma (CHOL) versus normal tissue. (B) Volcano plot of differential gene expression in kidney renal clear cell carcinoma (KIRC) versus normal tissue. (C) Volcano plot of differential gene expression in liver hepatocellular carcinoma (LIHC) versus normal tissue. GAS5 is highlighted in black color. Blue dots represent downregulated genes (∣Log2FC∣ > 0.8), red dots represent upregulated genes (∣Log2FC∣ > 0.8), and grey dots indicate genes without significant regulation (∣Log2FC∣ ≤ 0.8 and adjusted p ≥ 0.05)

We confirmed the expression level of GAS5 by retrieving publicly available microarray datasets from the GEO database. The differential expression analysis was performed with the GEO2R tool using the thresholds ∣Log2FC∣ > 0.8 and adjusted p < 0.05. DEGs were visualized to create volcano plots using the SRplot package (Figure 12). Further analysis revealed that our data showed significant upregulation of GAS5 in CHOL with the application of the GSE107943 dataset, which provided 30 samples of tumors and 27 adjacent tissues (Figure 12A, ∣Log2FC∣ = 2.04, adjusted p = 1.69×10−²⁸). In addition, the GSE213324 dataset of KIRC, which consists of 21 tumor samples and 20 adjacent tissues, shows significant upregulation (Figure 12B, ∣Log2FC∣ = 0.816, adjusted p = 0.00463) of GAS5. Moreover, in LIHC, the GSE46408 dataset, which collected samples of tumors and their matched normal tissues, also revealed a clear upregulation of GAS5 (Figure 12C, ∣Log2FC∣ = 1.86, adjusted p = 0.0124). All these findings point out with great consistency that GAS5 is significantly overexpressed in such malignancies and may contribute greatly to the pathogenesis process.

Insights into GAS5 expression and radiation therapy response

**Figure 13:**
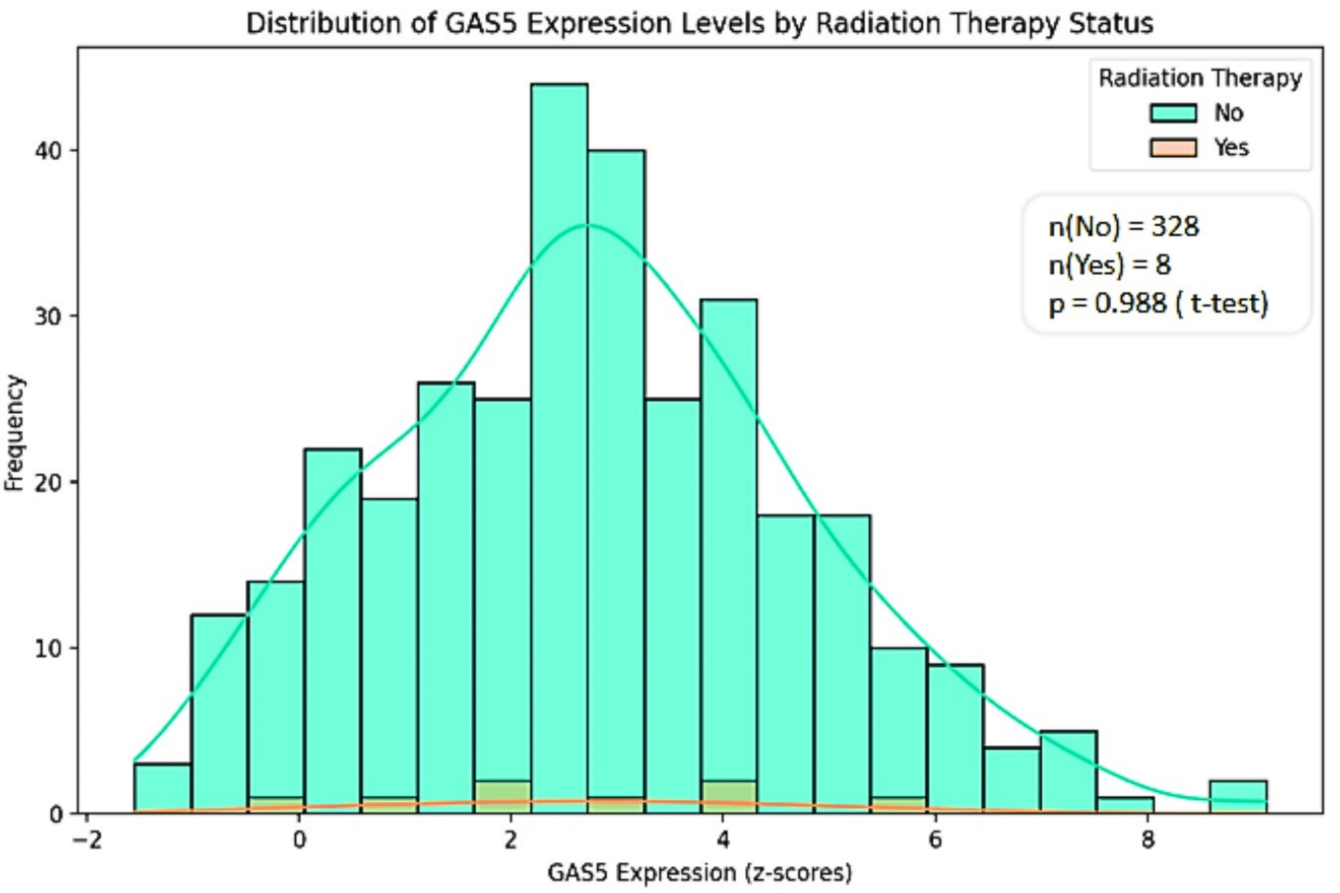
Distribution of GAS5 expression levels by radiation therapy status in LIHC using cBioPortal database GAS5: growth arrest-specific transcript 5 The histogram shows the distribution of GAS5 expression levels (z-scores) stratified by radiation therapy status ("Yes" and "No") in liver hepatocellular carcinoma (LIHC). The x-axis represents GAS5 expression levels, and the y-axis shows the frequency of samples. The "No" group (n = 328) is represented in green, and the "Yes" group (n = 8) is represented in orange. Statistical analysis revealed no significant difference between the groups (p > 0.05)

**Figure 14:**
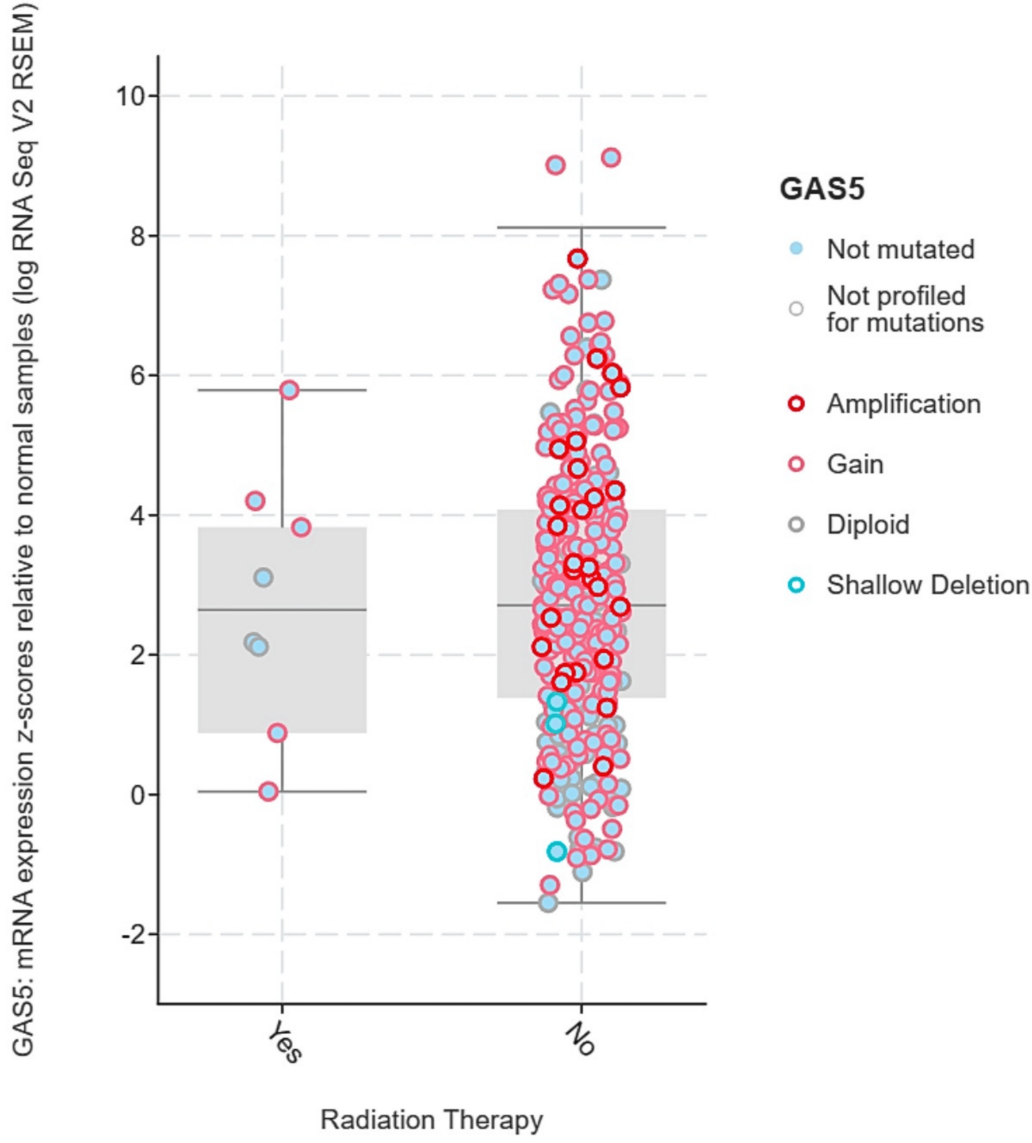
GAS5 expression levels by genetic alterations and radiation therapy status in LIHC using cBioPortal database GAS5: growth arrest-specific transcript 5 The box plot shows GAS5 expression levels (z-scores) stratified by genetic alterations (amplification, gain, diploid, and shallow deletion) and radiation therapy status ("Yes" and "No") in liver hepatocellular carcinoma (LIHC). The x-axis represents radiation therapy status, and the y-axis shows GAS5 expression levels. Genetic alterations are color-coded: amplification (red), gain (pink), diploid (gray), and shallow deletion (blue). Amplification and gain are more frequent in the "No" group and correlate with higher GAS5 expression levels, while diploid and shallow deletion are associated with lower expression levels

We analyzed the expression levels of GAS5 mainly in LIHC because of the availability of radiation therapy data from the TCGA Pan-Cancer Atlas dataset accessed via cBioPortal. GAS5 expression levels were stratified by radiation therapy status ("Yes" and "No") and categorized by genetic alterations, including amplification, gain, diploid, and shallow deletion. No significant statistical difference in terms of GAS5 expression levels between radiation therapy groups (p > 0.05) was established using the t-test and Mann-Whitney U test. However, there is a wider range regarding expression levels in the "No" group (z-scores: -1.5 to 8) as opposed to the "Yes" group (z-scores: 0 to 6) (Figure 13). Then, GAS5 gene expression levels were analyzed for genetic alterations in relation to radiation therapy (Figure 14). Amplification and gain were more frequent in the "No" group and had higher GAS5 expression levels. In contrast, diploid and shallow deletion changes were less frequent and associated with lower expression levels in both groups. However, we noted the unequal sample sizes between groups, with only eight in the "Yes" group compared to 328 in the "No" group for LIHC. Moreover, insufficient data were available for KIRC and CHOL to obtain similar conclusions. These results indicate possible associations between GAS5 genetic changes and radiation treatment response in LIHC, although the limited sample size in the irradiated group calls for further investigations in larger groups.

Coexpression network analysis of GAS5

**Figure 15:**
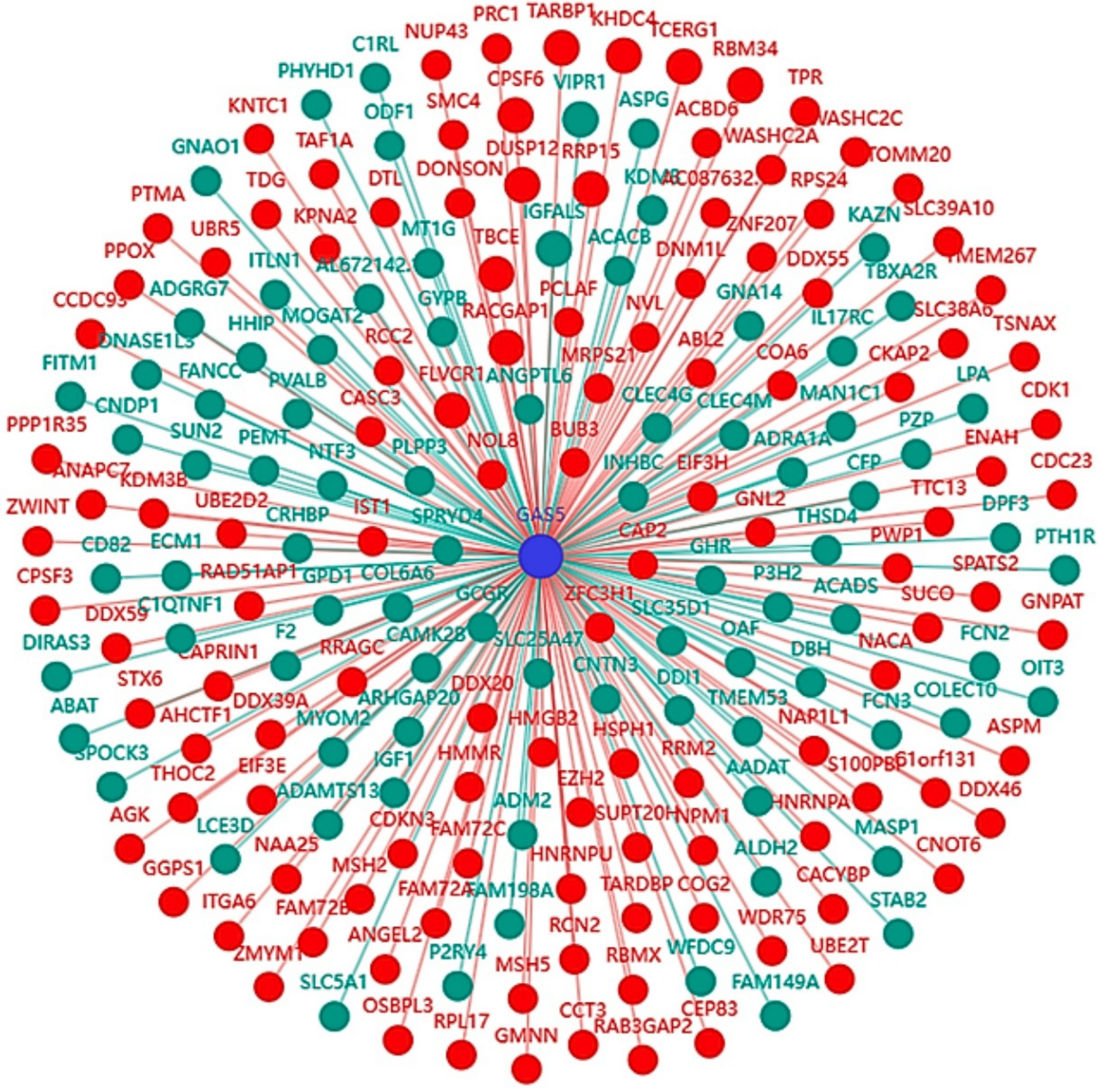
GAS5 coexpression network of GAS5 and associated mRNAs in LIHC GAS5: growth arrest-specific transcript 5 The network visualization shows correlations between GAS5 (central blue node) and 200 associated messenger RNA (mRNA) genes in liver hepatocellular carcinoma (LIHC), based on the LI_S532 study with a logFC of 1.5670 from the InCAR database. Red nodes indicate positive correlations, while green nodes indicate negative correlations with GAS5. Edge lengths are proportional to correlation strength, and gene symbols are labeled for each node. The radial layout highlights the interconnectivity of GAS5-associated genes

**Figure 16:**
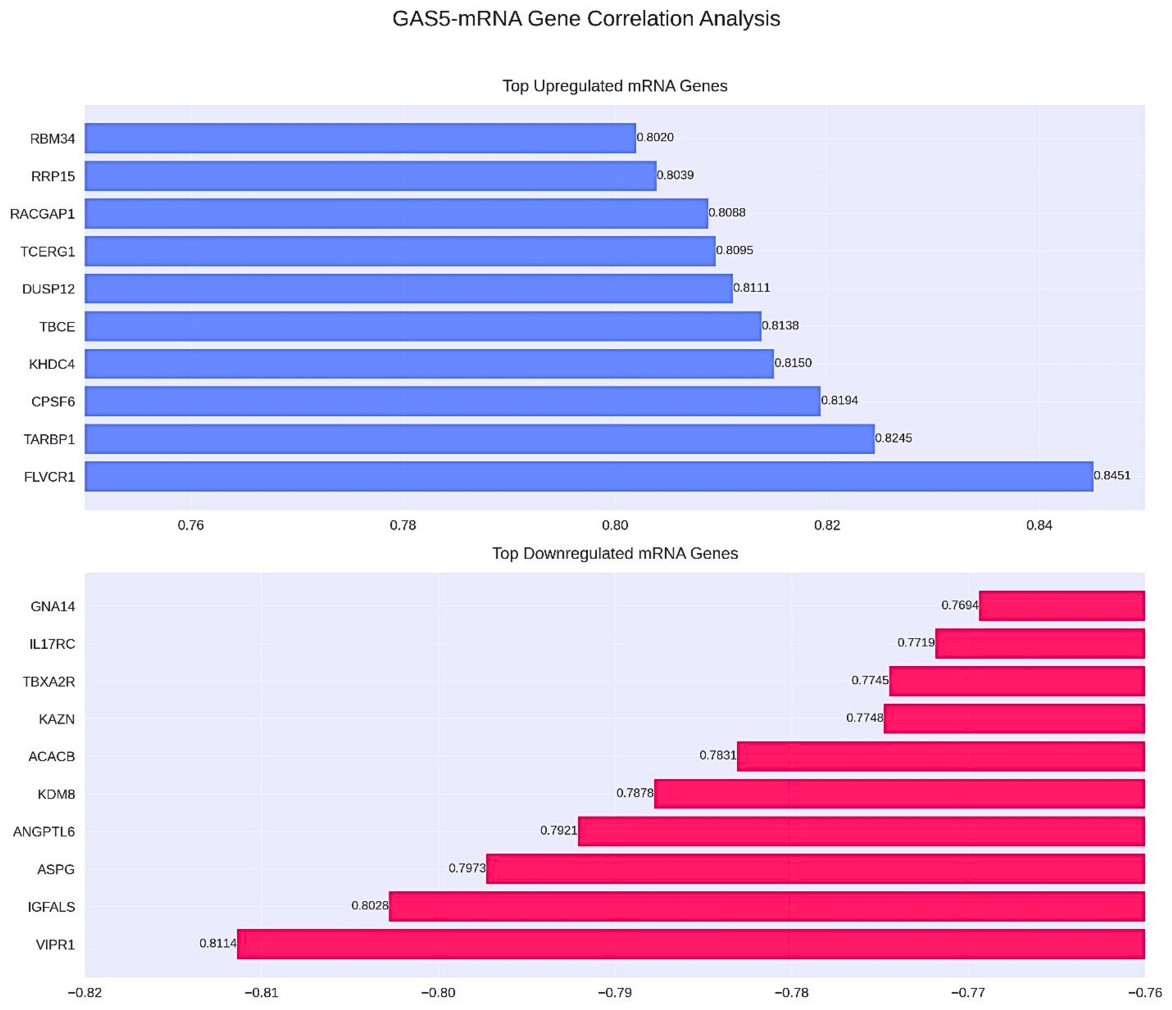
GAS5-mRNA gene expression correlation analysis: upregulated and downregulated genes in LIHC GAS5: growth arrest-specific transcript 5 The bar chart illustrates the regulatory relationship between long noncoding RNA GAS5 and its associated messenger RNA (mRNA) targets in liver hepatocellular carcinoma (LIHC), based on the LI_S532 study with a logFC of 1.5670 from the InCAR database. Horizontal bars represent Pearson correlation coefficients (r) between GAS5 and individual mRNA genes. The upper panel highlights the top 10 positively correlated genes. The lower panel displays the top 10 negatively correlated genes. Blue bars indicate positive correlations, while red bars represent negative correlations

The coexpression network analysis showed genes significantly coexpressed with GAS5 in LIHC (Figure 15). Among the 200 analyzed mRNA genes, 12 exhibited strong correlations (|r| ≥ 0.8), including 10 with strong positive correlations (r ≥ 0.8) and two with strong negative correlations (r ≤ -0.8), representing 6% of total interactions. The strongest positive correlations were with FLVCR1 (r = 0.845), TARBP1 (r = 0.825), and CPSF6 (r = 0.819), and the strongest negative correlations were seen in VIPR1 (r = -0.811) and IGFALS (r = -0.803, Figure 16). The comparisons of KIRC and CHOL were not possible due to data unavailability. Such an expression pattern suggests a possible regulatory function of GAS5 in pathways including these genes in LIHC.

The KEGG pathway enrichment analysis of GAS5 

**Figure 17:**
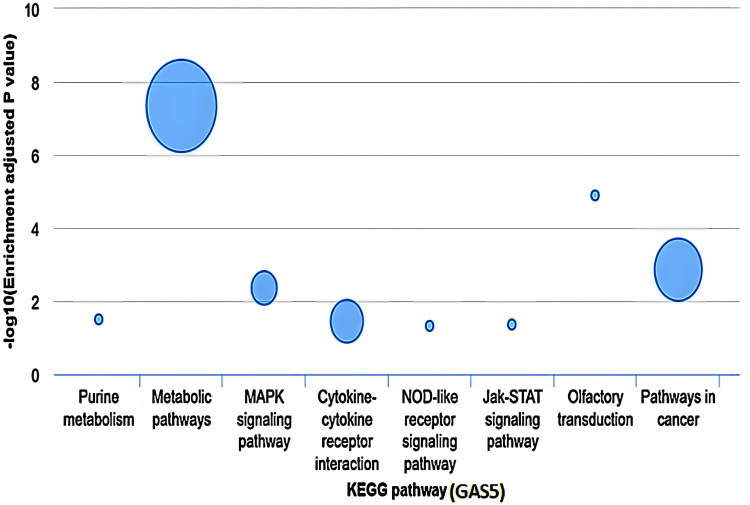
Enrichment analysis of KEGG pathways associated with GAS5 in LIHC GAS5: growth arrest-specific transcript 5; KEGG: Kyoto Encyclopedia of Genes and Genomes The bar chart illustrates the KEGG pathway enrichment analysis of GAS5-associated genes in liver hepatocellular carcinoma (LIHC), based on the LI_S532 study with a logFC of 1.5670 from the InCAR database. The x-axis represents the -log10(P) values, indicating the statistical significance of each pathway, while the y-axis lists the pathway names. Bubble size corresponds to the number of genes involved in each pathway, with larger bubbles indicating greater gene involvement

The KEGG pathway enrichment analysis revealed that GAS5 was significantly associated with several cellular pathways in LIHC (Figure 17). The most enriched pathway was metabolic pathways (−log10(P) ≈ 7) with 14 associated genes. Pathways in cancer showed moderate enrichment (−log10(P) ≈ 3) with six associated genes. MAPK signaling pathway and cytokine-cytokine receptor interaction had similar levels of enrichment (−log10(P) ≈ 2) with two and three associated genes, respectively. Other pathways, including purine metabolism, NOD-like receptor signaling, Jak-STAT signaling, and olfactory transduction, showed different enrichment levels. Notably, the olfactory transduction showed a higher enrichment level (−log10(P) ≈ 5) with one involved gene. The other pathways suggested low enrichment (−log10(P) < 2), and each pathway only had one involved gene. Nevertheless, this database did not indicate which specific genes contributed to each of the pathways, and further analysis is needed to explain the functional significance of GAS5. Data of KIRC and CHOL was not available for similar analyses.

GAS5 as a ceRNA

**Figure 18:**
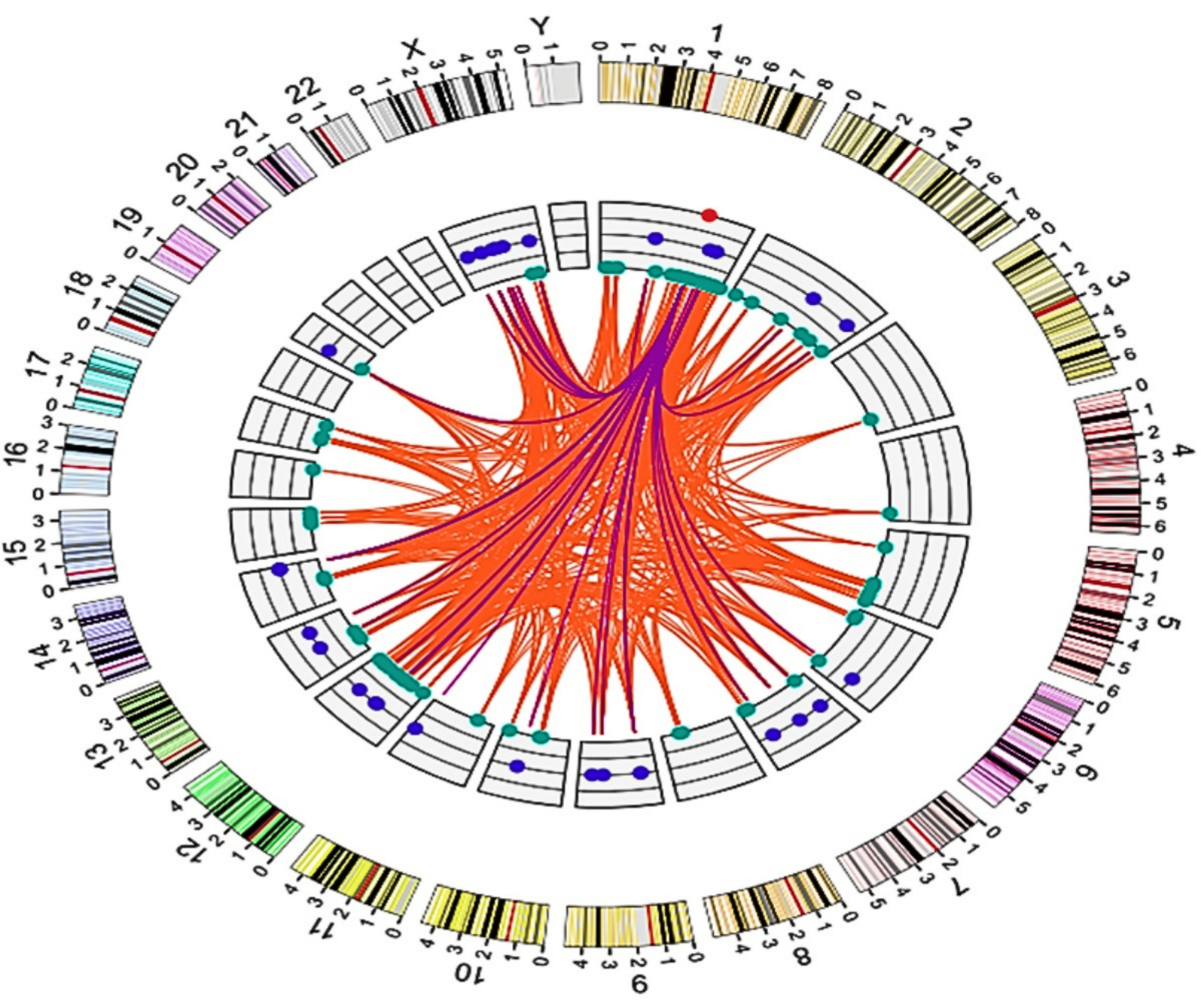
GAS5-linked competing endogenous RNA network in LIHC GAS5: growth arrest-specific transcript 5 The circular diagram depicts GAS5 (red dot) as a competing endogenous RNA (ceRNA) network in liver hepatocellular carcinoma (LIHC), based on the LI_S532 study with a logFC of 1.5670 from the InCAR database. The outer ring shows chromosomal regions, while the inner circle features nodes for microRNA (miRNA) binding sites (blue dots) and protein-coding genes (green dots). Orange lines illustrate the interactions among these elements, emphasizing the complex regulatory relationships involving GAS5

**Figure 19:**
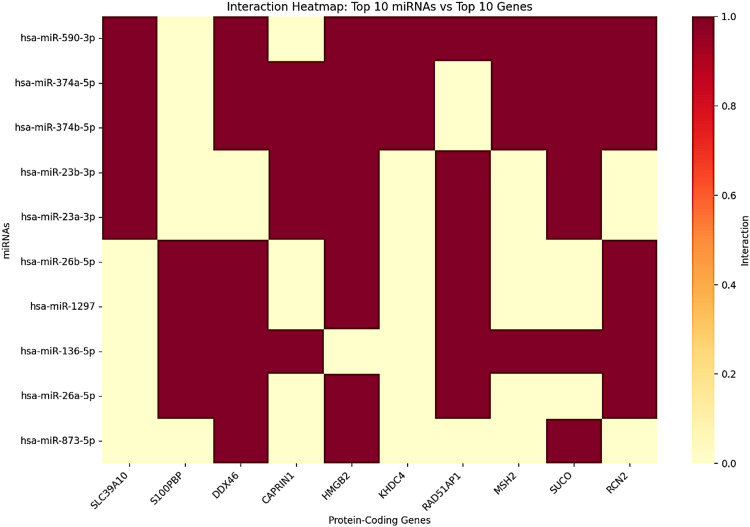
Interaction heatmap of the top 10 miRNAs and associated protein-coding genes in the GAS5-mediated ceRNA network in LIHC GAS5: growth arrest-specific transcript 5 The color intensity represents interaction strength of the microRNAs (miRNAs), with dark red indicating strong interactions and light yellow indicating weak or no interactions in liver hepatocellular carcinoma (LIHC). This heatmap is based on the LI_S532 study with a logFC of 1.5670 from the InCAR database

The lncRNA-miRNA-coding gene interaction network analysis identified GAS5 as a crucial ceRNA in LIHC. An integrated network analysis identified 674 unique regulatory pairs consisting of 36 miRNAs and 95 protein-coding genes (Figure 18). Among the top 10 miRNAs identified in the interaction network (Figure 19), has-miR-590-3p was the most connected node, targeting 57 protein-coding genes (8.46% of all interactions). It was followed by has-miR-374a-5p and has-miR-374b-5p, both targeting 38 and 36 genes, respectively. The rest of the top miRNAs were has-miR-23b-3p, has-miR-23a-3p, has-miR-26b-5p, has-miR-1297, has-miR-136-5p, has-miR-26a-5p, and has-miR-873-5p, suggesting a hierarchical organization of miRNA-mediated regulation within the network.

**Figure 20:**
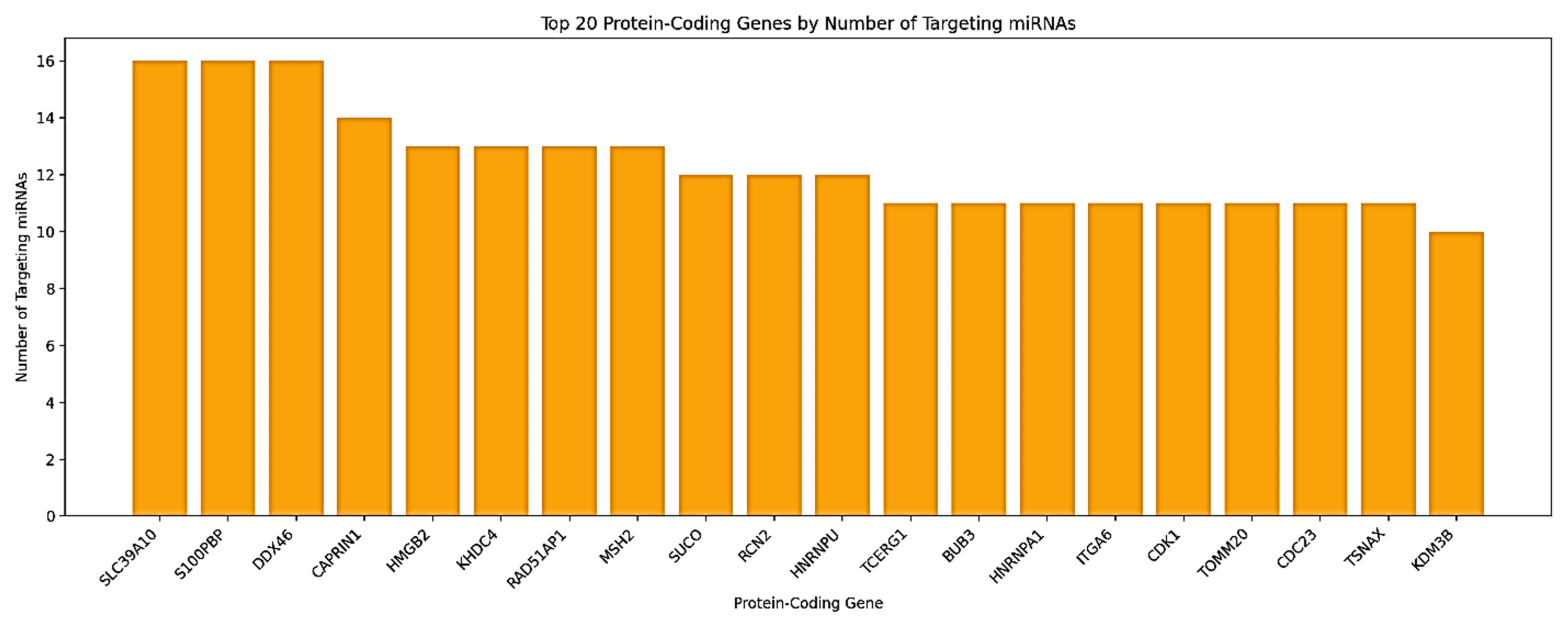
Distribution of miRNA targeting across the top 20 protein-coding genes in the GAS5-mediated ceRNA network in LIHC GAS5: growth arrest-specific transcript 5 The bar chart illustrates the number of microRNAs (miRNAs) targeting the top 20 protein-coding genes in the GAS5-mediated ceRNA network in liver hepatocellular carcinoma (LIHC), based on the LI_S532 study with a logFC of 1.5670 from the InCAR database. The x-axis represents the protein-coding genes, while the y-axis indicates the number of targeting miRNAs

Target gene analysis identified the most associated protein-coding genes with significant miRNA regulation (Figure 20). The highest-regulated genes were SLC39A10, S100PBP, and DDX46, each targeted by 16 distinct miRNAs. CAPRIN1 and HMGB2 were also among the highly regulated genes, each targeted by 14 and 13 miRNAs, respectively. Other important target genes included KHDC4, RAD51-associated protein 1 (RAD51AP1), MutS Homolog 2 (MSH2), SLCO3A1, and RCN2. Notably, paralogous miRNAs showed a surprising functional overlap (Table 1), which was exemplified unequivocally by the identical targeting patterns of hsa-miR-374a-5p and hsa-miR-374b-5p for genes such as SLC39A10 and HNRNPA1. These findings are indicative that GAS5 may function as a hub regulator in ceRNA networks and modulate critical oncogenic and tumor-suppressive pathways in LIHC. Regrettably, KIRC and CHOL data were not available to further explore and enable cross-cancer interpretation. 

**Table 1:** Summary of miRNAs and their associated coding genes in LIHC GAS5: growth arrest-specific transcript 5 The table presents the distribution of 36 microRNAs (miRNAs) and their corresponding number of protein-coding gene targets in the GAS5-mediated ceRNA network in liver hepatocellular carcinoma (LIHC), based on the LI_S532 study with a logFC of 1.5670 from the InCAR database. The first column lists the miRNAs, while the second column indicates the number of protein-coding genes targeted by each miRNA

No.	miRNAs	Coding genes
1	has-miR-128-3p	14
2	has-miR-1297	24
3	has-miR-135a-5p	20
4	has-miR-135b-5p	19
5	has-miR-136-5p	24
6	has-miR-137	19
7	has-miR-18a-5p	10
8	has-miR-18b-5p	9
9	has-miR-196a-5p	14
10	has-miR-196b-5p	13
11	has-miR-205-5p	13
12	has-miR-221-3p	20
13	has-miR-222-3p	16
14	has-miR-223-3p	13
15	has-miR-23a-3p	30
16	has-miR-23b-3p	31
17	has-miR-23c	10
18	has-miR-26a-5p	24
19	has-miR-26b-5p	28
20	has-miR-29a-3p	19
21	has-miR-29b-3p	21
22	has-miR-29c-3p	21
23	has-miR-3167	1
24	has-miR-346	4
25	has-miR-361-5p	17
26	has-miR-374a-5p	38
27	has-miR-374b-5p	36
28	has-miR-433-3p	21
29	has-miR-4465	9
30	has-miR-455-5p	8
31	has-miR-4735-3p	2
32	has-miR-485-5p	9
33	has-miR-544a	23
34	has-miR-590-3p	57
35	has-miR-873-5p	23
36	has-miR-876-5p	13

## Discussion

This study provides a comprehensive pan-cancer analysis of GAS5, revealing its multifaceted roles as a diagnostic, prognostic, and therapeutic biomarker across different malignancies. GAS5 has been considered a tumor suppressor; it regulates the cell cycle by keeping cells at the G0/G1 phase, inhibits cell proliferation, and induces apoptosis; all these mechanisms contribute together to inhibit cancer development [5,7]. Our findings contradict this concept by illustrating its context-dependent function and dual expression patterns. As evidenced by analyses from the TIMER, GEPIA, and UALCAN genomic databases, GAS5 was substantially upregulated in CHOL, KIRC, and LIHC. Consistent with our current discoveries regarding overexpression in these cancer types, prior research has demonstrated that GAS5 functions as an oncogene when expressed in specific conditions or contexts. In one previous study [21], GAS5 expression in CHOL was associated with advanced clinical stages and lymph node metastasis and showed potential in promoting proliferation and invasion by sponging miRNA mechanisms, particularly through the miR-1297 axis. In KIRC and LIHC, this relationship was even more complex, as GAS5 was generally upregulated, although its expression correlated with poor overall survival, indicating possible context-dependent functionality depending on hypomethylation or regulatory interactions [22,23]. These research findings uphold ours and show that GAS5 may be a diagnostic biomarker for CHOL, KIRC, and LIHC. It also seems to act more as a prognostic biomarker for LIHC and, to some smaller degree, for KIRC, which has only been supported by data coming from a single database. The variation of its prognostic value in different databases underlines the need for larger cohorts and standardized methodologies to emphasize its tissue-specific roles.

Our analysis showed clear patterns of GAS5 expression, with a strong association for cancer progression in KIRC but not in all stages of CHOL and LIHC. Notably, demographic stratification showed that male KIRC patients constantly displayed higher levels of GAS5 than females, with further variation across age groups and racial backgrounds. These patterns of expression display tissue-specific molecular contexts clearly. In the context of LIHC, GAS5 is positively correlated with immune cell infiltration, in particular B cells, CD4+ T cells, CD8+ T cells, and macrophages, which may indicate a role in modulating immune responses. In contrast, in KIRC, GAS5 shows a negative correlation with immune cell infiltration, particularly for B cells and dendritic cells, suggesting possible immunosuppressive roles. Among the prognostic biomarkers identified for LIHC and KIRC, such as bone marrow kinase (BMX) [24], GAS5 distinguishes itself through its upregulation and context-dependent modulation of the tumor-immune microenvironment [23]. While low BMX expression is associated with poor prognosis in LIHC without significant immune modulation, GAS5 plays a more active role by influencing immune cell infiltration and regulating pathways like MAPK signaling through its ceRNA activity. These findings provide a clear rationale for taking into account both individual genomic profiles and tissue-specific molecular contexts in the development of targeted therapeutic strategies.

In contrast, GAS5 shows downregulation in some other types of cancers, such as breast cancer. A previous study [25] showed that GAS5 downregulation is associated with higher TNM stages, lymph node metastases, poor overall survival, and increased resistance to chemotherapy. Moreover, GAS5 acts as a tumor suppressor in breast cancer through modulation of the PI3K/AKT/mTOR pathway and participation in Wnt/β-catenin signaling. It thus delays the advancement of pathways important for the regulation of cell-cycle progression, cell survival, and metabolic processes. In addition, GAS5 also sponges miR-221-3p, which downregulates DKK2, an essential inhibitor of the Wnt/β-catenin pathway, specifically in triple-negative breast cancer (TNBC). It is observed that mTOR and GAS5 suppression mutually regulate each other, thereby creating a complicated feedback loop [25]. Interestingly, GAS5 was highly upregulated in metastatic SKCM compared to nonmetastatic tumors. Although it usually acts as a tumor suppressor by inhibiting migration and invasion by regulating MMP2 and MMP9, its overexpression in metastatic cases indicates either a compensatory mechanism or context-specific functionality [26].

Epigenetic regulation has been identified as an important mechanism in GAS5 expression, and our analysis showed a consistent pattern of hypomethylation in CHOL, KIRC, and LIHC. The current study is in line with previous findings [23], which also showed that CpG site hypomethylation of GAS5 is associated with upregulated expression in KIRC and LIHC, related to increased transcriptional activity, and with a tendency to poor prognosis. These findings support the fact that epigenetic changes modulate GAS5 expression and function and may, therefore, open therapeutic avenues by epigenetic modulation [25,26]. These variable expression patterns of GAS5 in different tumor types underline the context-dependent functionality, determined by the tumor microenvironment and complex regulatory networks like miRNA sponging, chromatin remodeling, and transcription factor binding. For example, GAS5 acts as a tumor suppressor in hepatocellular carcinoma by inhibiting migration and invasion through modulating miR-21 [27], while in cholangiocarcinoma, it has a promoting effect by regulating hsa-miR-1297 [21]. Such tissue-specific behavior shows that there should be a pan-cancer view to understand the full biological function and clinical implications of GAS5.

Therapeutic implications of GAS5 in cancer

Recent advances in the understanding of the role played by GAS5 in cancer have shown tremendous therapeutic potential, especially in overcoming resistance to therapy and increasing treatment efficacy. Low expression of GAS5 has been demonstrated to be one of the major determinants of chemotherapy resistance, especially in cervical cancer, where low GAS5 expression is associated with poor survival and resistance to cisplatin. The mechanism involves GAS5 functioning as a ceRNA by sequestering miR-21, thus upregulating the expression of pro-apoptotic genes, including PDCD4, and sensitizing cancer cells to cisplatin treatment [28]. GAS5 in radiation therapy holds great promise to improve the outcomes in treatment, and in the case of breast cancer, it sensitizes cells to ionizing radiation by inhibiting miR-21-mediated DNA repair pathways [9]. In hepatocellular carcinoma, GAS5 has been found to enhance radiosensitivity via sponging miR-144-5p, which subsequently upregulates the expression of ATF2. Overexpression of GAS5 increases levels of ATF2, hence increasing radiosensitivity, while knockdown of GAS5 restrains proliferation and facilitates radioresistance [29]. Our network analysis identified GAS5 as a key regulatory hub, acting as a ceRNA, regulating interactions involving 36 miRNAs and 95 protein-coding genes in LIHC. These have been implicated in the regulation of key cellular pathways, especially those relevant to metabolic processes and tumor development. Notably, we found several key genes, including RAD51AP1, MSH2, cyclin-dependent kinase 1 (CDK1), and HMGB2, known to be involved in the DNA damage response and sensitivity to radiation. Especially, HMGB2 seems to be the central controller involved in the DNA damage response to radiation, promoting proliferation and invasion of cancer cells and regulating chromatin structure; targeting the GAS5 regulatory network could therefore potentially result in an overall enhanced effectiveness of treatment [30]. In summary, GAS5 epigenetic regulation offers an opportunity for therapeutic intervention; some therapeutic agents, like the DNA methylation inhibitor 5-aza-2′-deoxycytidine (5-aza-dc), have been used in some preclinical studies on melanoma to restore the expression of GAS5 and thereby suppress tumor development [26]. Understanding the tissue-specific functions and regulatory networks of GAS5 is essential for advancing therapeutic strategies, such as mimetics of GAS5 and RNA-based treatments targeting the miRNA pathways, including antisense oligonucleotides. These strategies have the potential to enhance treatment efficacy by modulating key oncogenic pathways. Integrating GAS5-targeted therapies with existing treatments, such as chemotherapy or radiotherapy, offers a promising avenue for improving therapeutic outcomes and advancing precision medicine across diverse cancer types.

Study limitations 

This analysis is based on publicly available genomic datasets that may not always be representative of the whole landscape of cancers and patient populations. In addition, the sparse data available for some cancers, especially CHOL and KIRC, were limiting in cross-cancer comparisons. Moreover, the generally small sample sizes of subgroup analyses, especially those based on demographic features or radiation therapy status, highlight the importance of standardized methodologies and an increased cohort size for reliable validation. Future studies on GAS5 should focus on confirming its role in cancer by using laboratory studies, including in vitro and in vivo experiments. This can validate its role in tumor progression, immune regulation, and resistance to therapy. Moreover, it is necessary to extend the analyses to those cancers that are underrepresented to better understand their context-specific functions and clinical application potential.

## Conclusions

This pan-cancer analysis identified GAS5 as a multifunctional lncRNA with deep implications in cancer biology. Our findings establish its potential as a diagnostic biomarker, especially in CHOL, KIRC, and LIHC, and highlight its prognostic value, particularly in LIHC, with additional relevance in KIRC. We found tissue-specific expression patterns and notable associations with immune cell infiltration and genetic alterations, indicating that molecular context should be considered in clinical applications. Moreover, we highlighted in LIHC the links associated with GAS5 and pathways of relevance, especially those that may confer radiosensitivity and its role within the ceRNA network, suggesting promising therapeutic opportunities, such as enhancing radiotherapy efficacy or developing targeted epigenetic therapies. These findings would help pave the way for future research to validate GAS5-targeted therapies in preclinical models and clinical trials.

## Appendices

**Table 2:** List of abbreviations

Abbreviation	Definition
5-aza-dc	5-aza-2'-deoxycytidine
AJCC	American Joint Committee on Cancer
AKT	AKT serine/threonine kinase
ANOVA	Analysis of variance
ATF2	Activating transcription factor 2
BMX	Bone marrow kinase
BRCA	Breast invasive carcinoma
CDK1	Cyclin-dependent kinase 1
CHOL	Cholangiocarcinoma
COAD	Colon adenocarcinoma
CPSF6	Cleavage and polyadenylation specific factor 6
DEGs	Differentially expressed genes
EMT	Epithelial-mesenchymal transition
ESCA	Esophageal carcinoma
FLVCR1	Feline leukemia virus subgroup C cellular receptor 1
FPKM	Fragments per kilobase million
GAS5	Growth arrest-specific transcript 5
GEO	Gene Expression Omnibus
GEPIA	Gene Expression Profiling Interactive Analysis
GTEx	Genotype-Tissue Expression
HCC	Hepatocellular carcinoma
HMGB2	High mobility group box 2
IGFALS	Insulin-like growth factor binding protein, acid labile subunit
KEGG	Kyoto Encyclopedia of Genes and Genomes
KICH	Kidney chromophobe
KIRC	Kidney renal clear cell carcinoma
KIRP	Kidney renal papillary cell carcinoma
LIHC	Liver hepatocellular carcinoma
LUAD	Lung adenocarcinoma
LUSC	Lung squamous cell carcinoma
MAPK	Mitogen-activated protein kinase
MSH2	MutS Homolog 2
NK	Natural killer
NSCLC	Non-small cell lung carcinoma
OS	Overall survival
PDCD4	Programmed cell death 4
PI3K	Phosphoinositide 3-kinase
PRAD	Prostate adenocarcinoma
RAD51AP1	RAD51-associated protein 1
READ	Rectum adenocarcinoma
SKCM	Skin cutaneous melanoma
STAD	Stomach adenocarcinoma
TARBP1	TAR (HIV-1) RNA-binding protein 1
TCGA	The Cancer Genome Atlas
THCA	Thyroid carcinoma
TIMER	Tumor Immune Estimation Resource
TNBC	Triple-negative breast cancer
TPM	Transcripts per million
UALCAN	University of Alabama at Birmingham Cancer Data Analysis Portal
UCEC	Uterine corpus endometrial carcinoma
VIPR1	Vasoactive intestinal peptide receptor 1
lnCAR	Long noncoding RNA cancer arrays
lncRNA	Long noncoding RNA
mRNA	Messenger RNA
mTOR	Mechanistic target of rapamycin kinase
miRNA	MicroRNA
